# SensoTube: A Scalable Hardware Design Architecture for Wireless Sensors and Actuators Networks Nodes in the Agricultural Domain

**DOI:** 10.3390/s16081227

**Published:** 2016-08-04

**Authors:** Dimitrios Piromalis, Konstantinos Arvanitis

**Affiliations:** 1 Department of Natural Resources Management and Agricultural Engineering, Agricultural University of Athens, Iera Odoos 75, Athens 11855, Greece; karvan@aua.gr; 2 Department of Automation Engineering, Piraeus University of Applied Sciences (TEI of Piraeus), P. Ralli and Thivon 250, Egaleo 12244, Greece

**Keywords:** wireless, sensors, actuators, networks, open-source, expandable platforms, Arduino, ARM, energy management, agriculture

## Abstract

Wireless Sensor and Actuators Networks (WSANs) constitute one of the most challenging technologies with tremendous socio-economic impact for the next decade. Functionally and energy optimized hardware systems and development tools maybe is the most critical facet of this technology for the achievement of such prospects. Especially, in the area of agriculture, where the hostile operating environment comes to add to the general technological and technical issues, reliable and robust WSAN systems are mandatory. This paper focuses on the hardware design architectures of the WSANs for real-world agricultural applications. It presents the available alternatives in hardware design and identifies their difficulties and problems for real-life implementations. The paper introduces SensoTube, a new WSAN hardware architecture, which is proposed as a solution to the various existing design constraints of WSANs. The establishment of the proposed architecture is based, firstly on an abstraction approach in the functional requirements context, and secondly, on the standardization of the subsystems connectivity, in order to allow for an open, expandable, flexible, reconfigurable, energy optimized, reliable and robust hardware system. The SensoTube implementation reference model together with its encapsulation design and installation are analyzed and presented in details. Furthermore, as a proof of concept, certain use cases have been studied in order to demonstrate the benefits of migrating existing designs based on the available open-source hardware platforms to SensoTube architecture.

## 1. Introduction

Wireless Sensors and Actuators Networks (WSANs) is an established and challenging technology, with a great potential impact on the measurement, communication and control applications to a variety of activities of the modern postindustrial society. According to market analyses, WSAN node sales will constitute a multi-trillion market in the next few years [1,2]. Given the fact, during the last 25 years, the agricultural production sector has been transformed from a traditional labor-intensive sector into a technology-intensive one, it has been strongly considered as a very prosperous potential area for WSAN technology use. Indeed, a vast range of existing and future WSAN applications in agriculture have been identified and reported by many researchers [3,4,5]. Moreover, relatively-new terms have been introduced in current terminology, in order to express the trends in modern agriculture, such as: precision agriculture; precision farming; variable-rate management; etc. On the other hand, the majority of WSAN deployments in agriculture are taking place on a short-scale research and development basis, rather than on a large-scale commercial solutions basis. The massive expansion of WSAN technology in agriculture appears to lag behind the market’s expectations. Many researchers have reported certain technological causes for this, which relate to hardware design issues, such as standardization in protocols and development tools, configurability, expandability, scalability in computing power, and memory capacity [3,6,7,8,9]. 

Examining the idiosyncrasy of the real-world (real-life) WSAN applications, the most common critical features found are: the operation in the real environmental and spatiotemporal conditions (far away from the lab), the systems are on the end-users hands, the energy autonomy, the need for long-term serviceability and management, the maintainability, the expandability, the reconfigurability, the reusability, the robustness and ruggedness, the long-standing reliability and the low cost per WSAN node [10]. Therefore, any single factor, which can negatively affect one or more of the aforementioned features, could jeopardize the success of the whole WSAN implementation. Regarding agriculture, these features have greater impact to the success of WSAN applications, because, traditionally, this sector requires scalable wireless networks comprised by large number of nodes covering huge physical remote areas, capable of measuring a variety of physical parameters [11]. Furthermore, very often, the harsh operating environment has been reported, by many experts, as a major source of implementation problems [12,13]. Extreme temperatures, humidity, rain, snow, wind, and sunlight radiation, can all seriously threat the normal operation of WSAN hardware systems, which, in their vast majority, have been designed for indoor environments (labs, offices, etc.) [6,9,14,15,16]. Unfortunately, the physical agricultural environment cannot successfully be modeled and tested by simulation methods (e.g., [17]). For example, the behavior of real batteries [18,19] or the radio signal propagation [20,21] are typical cases of operation parameters that are extremely difficult to model and fully simulate, so the design of hardware WSAN systems for the agricultural domain is more complicated than in other application domains. 

**Figure 1:**
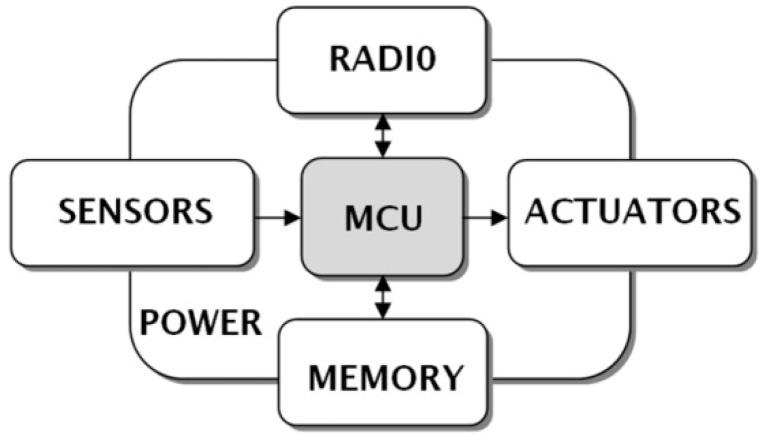
Typical architecture of a WSAN node.

Since the early days of the WSAN technology up to today, nodes’ hardware architecture was solely governed according to the block diagram depicted in Figure 1 [22,23]. It is a microcontroller-based system with all the necessary circuitry for sensors, and actuators, equipped with a radio frequency transceiver for data communication and networking. Even recent research attempts to use field-programmable gate arrays (FPGAs), instead of microcontroller units (MCUs), in WSAN nodes are based to the traditional architecture [24,25]. In general, such a node can be appropriately software configured to operate either as end-device, router, coordinator, or, with some extra modifications, as coordinator with gateway functionality. The role configuration is dictated by the networking protocol residing into the MCU’s program memory, in the form of a firmware stack. Moreover, there is a power source, commonly not implemented entirely on-board in order to cover the energy needs of the system’s components. Regarding the hardware design of the nodes, several realizations that have been introduced in the last fifteen years are strongly influenced by the doctrine of Smart Dust concept (miniaturization, etc.) [6,26]. The majority of them follow the typical architecture (Figure 1). In practice, all of these nodes enabled the research of WSAN systems in agriculture, because they were mainly used as the development tools, on the basis of which several conglomerate WSAN studies were conducted in the field. 

However, the solutions based on the traditional architecture are under question about their effectiveness in real-world WSAN implementations in agriculture, especially, in the light of the continuously changing technological environment, which is influencing this particular domain. From its infancy, WSAN has been a multi-facet technology. Although WSAN hardware solutions have not been ready enough, in order to accommodate the present requirements of real-life applications, new WSAN applications raise new limits for the hardware systems [27]. Indicatively, wireless multimedia networks (WMNs) [28,29,30]; sensor clouds [7]; integration of WSAN and radio frequency identification (RFID) systems [31]; collaboration of WSAN and satellite technology [32]; internet of things (IoT) [33]; and so on, are only some of the trends in modern WSAN applications. Further, with regard to the purely technological aspects of this changing environment, there has been an explosion of new challenging technologies that potentially could help towards the design of more reliable, robust and efficient WSAN systems. Among the most interesting of such technologies are energy harvesting and power management [34]; new networking protocols, such as the IEEE 802.15.4x, LoRa-Net, 6LoWPAN [35,36], DASH7 [37], etc.; big data and data fusion [13]; new human-machine-interfaces (HMI) powered by smart phones and portable computing and connectivity [9]; new micro-electromechanical sensors (MEMS) and advanced sensory concepts such as the Lab-on-Chip (LoC) [38]; new embedded processors such as the low energy and high performance ARM microcontrollers [39,40]; and, new energy storage media such as lithium-ion-based batteries and hybrid ultra-capacitors [41,42]. 

Nowadays, the WSAN hardware solutions, as described in [6], are classified into three classes, namely end-to-end solutions, generic solutions, and research solutions. Furthermore, according to the same study, there are three different WSAN nodes design spaces: the network space, the device space and the application space. Both the preceding and the contemporary nodes are far from meeting all the requirements of real-world applications. In order to ensure the success of WSAN implementations, there must be a close collaboration among the stakeholders, with the focus being on the application requirements. On the other hand, whatever the approach of the design space for WSAN hardware is, there are deficiencies in architectures openness, expandability, and configurability as well as complexities in implementation. 

As reported by many experts [3,6], in parallel to the lack of effective hardware design architectures, an additional significant barrier to successful systems development is the very long learning curves associated with several different aspects of the WSAN technology. This is the reason why a mix of multi-blended skills is necessary in research teams. A practical respond to overcome the know-how shortages, was the adoption of ready-made hardware solutions available in the market and their integration, in order to build final applications. This is commonly referred to as the commercial-of-the-shelf (COTS) approach. COTS are met either in the form of ready-made WSAN nodes, also known as motes, designed according to the traditional architecture [43], or in the form of specific-oriented testbeds. Admittedly, COTS-based systems design is an attractive approach for many practical reasons: it helps to reduce the development time, it absolves developers from re-inventing the wheel, it closes knowledge and skills gaps by providing ready-made resources (protocol stacks, etc.), it is relatively lower in cost compared to new prototype systems that suffer from high non-recurring engineering (NRE) costs [44,45,46,47]. However, the adoption of COTS approach precludes, in many cases, the deliberation of researchers and practitioners to extend their study and application areas. Furthermore, COTS-based solutions have not been designed in order to cope with the harsh external environmental conditions of specific applications, as in the case of agriculture. Therefore, this hardware design approach appears to be one-way, for building pilot and proof of concept units, but due to the limited expandability and reconfigurability imposed by various bounded architectures (in fact, slightly modified alternatives of the traditional architecture of Figure 1), it is incapable of supporting the requirements of real-world WSAN applications, especially in the agricultural domain [9,28,41,48].

WSANs’ developers in their persistent search of hardware platforms that allow for reconfigurability and expandability, have often recurred to generic open-source hardware (OSH, or OSHW) solutions such as the Arduino, Raspberry Pi and others [49,50,51]. Such platforms have been mainly created for embedded systems students and hobbyists. In particular, all of these solutions rely on the scenario of a MCU-based board that can be programmed using easy-to-learn C-language functions (through application peripheral interface (API) libraries) and are easily expanded using pass-through pin-headers by connecting additional application-specific boards. A huge number of ready-made firmware applications, examples and other programming resources have been produced and are freely available as open-source code from an extremely big developers’ community. This is very attractive for engineers and researchers who wish to overcome the limitations of embedded systems design and programming [3,41,52]. In agriculture there are already WSAN applications, where the aforementioned multi-board platforms have been used, either in cases of new system designa, or in cases of modifications to expand the functions of existing systems [9,14,49,53].

Summarizing, all the existing hardware design approaches, namely, the traditional architecture, the COTS-based approach, and the expandable OSH multi-boards platforms, appear to impose significant limitations and they inhibit the expansion of real-world WSAN implementations. Under these circumstances, the end-users in agriculture are very skeptical regarding the benefits-to-cost ratio. Robust and reliable WSAN systems tolerant of real-world applications needs, is a mandatory factor for WSAN credibility [8]. As it has been highlighted in [3], there is a lack of complete frameworks capable of allowing the development of systems from data acquisition to modeling and decision support. Therefore, it is obivious that the required new platforms should meet both the research needs and the needs entailed by real-life applications in the field of agriculture. 

This paper proposes the SensoTube, as a new open-source-oriented architecture for designing WSAN systems, with emphasis on the real-world implementations in the agricultural domain. SensoTube aims to overcome existing difficulties and problems in systems design and to provide, in a well-standardized and open way for the endeavored expandability, scalability, reconfigurability, reusability, testability, energy efficiency, and encapsulation needed in real-world WSAN applications. 

The rest of the paper is structured in eight sections, as follows: Section 2 presents the contemporary challenges and trends in WSAN node’s hardware design and focuses on the multi-board expandable architectures that are based on open-source-hardware approaches, due to their fundamental advantages. Moreover, several critical issues are identified in this section and they are categorized in five classes: signals management, power management, firmware development, programming and debugging and robustness and reliability. This section essentially aims at pointing out the reasons for which the existing expandable OSH architectures cannot be considered as being the best approach for real-life applications. Section 3 presents the proposed new scalable multi-tier architecture, namely the SensoTube, for WSAN hardware design. In particular, this section explains the concept of the proposed architecture and it provides the details of its tiers’ operation, with emphasis on real-world WSAN applications in the agricultural domain. The implementation reference model of the SensoTube architecture is reported in Section 4. In this section, the proposed mechanisms and methods for substantial boards’ expandability and control, in terms of signals, energy, communication, programming and debugging functions are also presented. Section 5 presents the alternatives that a SensoTube-based WSAN system can support for firmware and software development. Section 6 discusses the encapsulation approaches in WSAN deployments in the agricultural domain and it provides a critical analysis for the adoption of the tube-based encapsulation of the SensoTube architecture. Section 7 presents certain use cases in with real measurements and data of three popular open-source hardware platforms, and it demonstrates the benefits for migrating existing open-source designs to SensoTube architecture. Section 8 quotes the cost implications of the adoption of the proposed architecture. The paper is completed in Section 9, wherein the key benefits of adopting the SensoTube architecture for stakeholders are discussed and summarized together with future research directions. 

## 2. Challenges, Trends and Constraints in WSAN Hardware Design

Designing hardware systems for WSAN is always an arduous work. Knowledge, skills, and experience have to be demonstrated in fields such as digital and analog electronics, embedded systems, MCUs’ firmware development, power electronics, sensors, radio frequency (RF) communications, wireless networking, printed circuits board (PCB) design, prototyping, testing and evaluation. Furthermore, it is necessary to have a keen awareness of updated solutions launched by the microelectronics industry, which can benefit new systems’ designs (i.e., new integrated semiconductors (ICs), systems-on-chip [54] and other electronic components, in general). Diving deep into such fields is very often out of scope, for example, in cases where the aim is to monitor a particular physical phenomenon using WSAN technology in-situ. Although the aforementioned requirements are critical preconditions to WSAN systems, they are not included in the training courses for WSAN systems and applications [55]. The view perspectives of what is a WSAN system may vary among different areas of interest [6]. In particular, for the agricultural domain, the various differences in perspectives of a WSAN node are summarized in Table 1.

**Figure 2:**
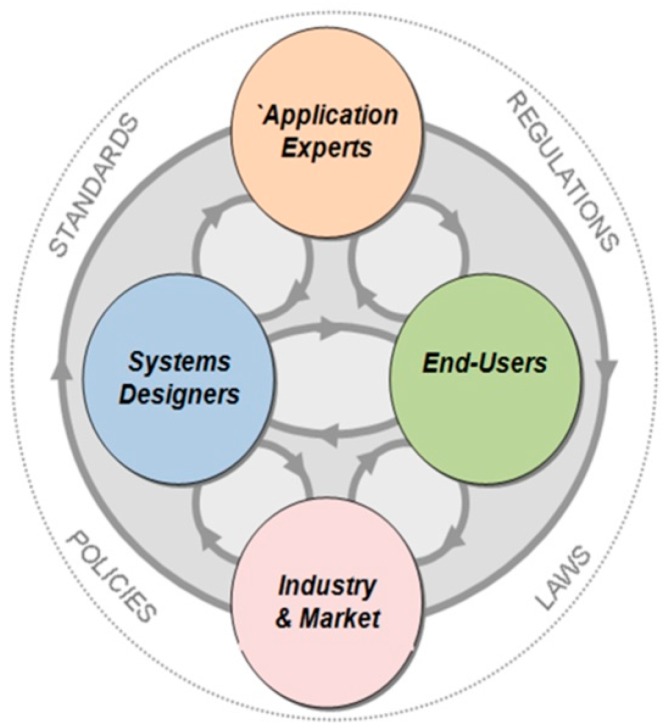
Interactions among different stakeholders in WSAN hardware systems design.

Design inefficiencies like the fragmentation and limitations caused by the lack of skills can jeopardize the anticipated full-scale commercialization and popularization of WSAN systems. In order to build successful systems that can face the difficulties and the requirements of real-life applications, each stakeholder has to take into consideration other stakeholders’ needs and idiocyncracies. As depicted in Figure 2, there are five major stakeholder groups, namely the application experts, the systems designers, the end-users, the industry/market, and the authorities (the external circle in Figure 2). The arrows illustrate the influence between different parts. Practically, this influence is based on the flow of tangibles (e.g., technologies, systems, tools, documents, etc.) and intangibles (e.g., skills, ideas, needs, expectations, etc.).

Obviously, the typical architecture of Figure 1 or the COTS approach cannot support the design of systems that will meet all the expectations of everyone that has a vested interest in a WSAN application. On the other hand, the reaction of the expandable multi-board systems’ designers and developers signals the direction for future architectures, in order to confront the changing, demanding, and complex applications’ ecosystems.

### 2.1. Embedded Systems Development Technologies and Market Trends

**Figure 3:**
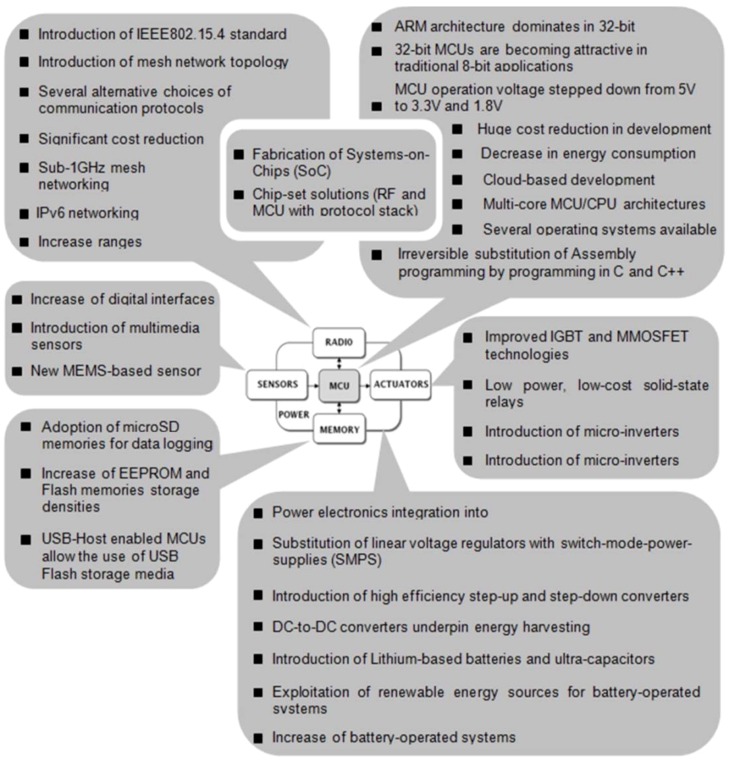
Technological trends and changes influencing the design of a WSAN hardware system.

Embedded systems technology has strongly been influenced by the dramatic changes that have occurred in the mobile phone market. In the last decade, consumer demand for ever more powerful smart phones have driven the electronics industry to design and manufacture high processing and low power MCUs and microprocessors (MPUs). This evolution has helped the introduction of mobile computing devices, e.g., tablets, etc., which in turn has acted as an additional reason for the development of new semiconductors, processors, sensors, batteries, and communication modules. Due to economies of scale of such markets, the cost of embedded systems has significantly diminished, whilst the cost-to-performance ratio has increased notably. Consequently, the design of hardware WSAN solutions has vastly been affected by the aforementioned changes. Ιn Figure 3, the most important changes in technologies and approaches associated with the sub-parts of the typical WSAN node system, in the last decade, is illustrated. Obviously, nowadays, experienced designers and developers have plenty of choices at their disposal, in order to build either end-to-end generic commercial solutions or optimized application-specific solutions. In particular, in the field of agriculture, there are stanch technologies for energy, communications, processing, etc. that can positively help towards the development of reliable and vigorous outdoor WSAN systems. Of course, the lack of skills and knowledge make these efforts difficult. 

On the other hand, this technological breakthrough brings about hurdles race conditions, because the revolutionary technologies have to be quickly assimilated and used, new development tools have to be launched to support designers in the previous effort as well as to produce new technology in their turn, whilst at the same time, under the pressure of stakeholders for robust and standardized solutions, the new solutions have to be commercialized as soon as possible. As a response to this perpetual need, many significant developments have been made by the electronics industry and market, in order to provide new tools, methods and solutions that encapsulate new technology and allow fast prototyping (such as the Mbed [56] and Codebender [57], and i-Sense [58]). The real revolution came from a new area of systems development, the so-called open-source software and hardware. According to this concept, the various design artifacts, i.e., documentation, circuits, software code, hardware project implementations, application case studies, etc. are freely shared among big users’ communities under the license scheme of Creative Commons Attribution Share-Alike, which allows for both personal and commercial derivative works as long as the credit to original creator is granted [59]. The most successful case of the open-source design approach is the Arduino platform [60]. 

The Arduino platform (Arduino SRL, Scarmagno, TO, Italy) is a MCU-based board using an Atmel AVR 8-bit MCU, which provides all of the microcontroller’s pins to pass-through pin-headers. Through these headers, all the major functional peripherals of the MCU are available to users. Users, in their turn, can connect other personal or commercial hardware boards, called shields, to the main Arduino board, in order to build their own specific applications. In order to make easy the firmware development process to users without much experience in embedded systems, Arduino provides a ready-made library of APIs in its integrated development environment (IDE). Thus, developers dispose an open-source hardware and software platform that allows the expandability and reusability they are looking for. Despite the fact that Arduino was originally established for education and hobbyists [61], it soon became very popular in research and development of real-life applications, even in demanding areas, such as agriculture. In recent years, the electronics industry realized the advantages of the open-source design approach and Arduino concept and it is foreseen as a new prosperous market. The idea of expandable modular hardware development tools and the application-centric programming concepts, of course, cannot be attributed to Arduino or to its successors. Many implementations, such as the Basic Stamp for MCU programming in Basic language at early 1990s [62,63], and the e-Blocks modular hardware tools [64], targeted providing easy-to-build hardware embedded systems. The reason that these efforts didn’t attract the popularity of Arduino concept is probably associated with the fact that they were single-source commercial solutions with negative cost and openness implications. The free support from a vast community of designers and developers, in the case of open-source platforms, has made the big difference, and it seems to be the solution to the way out of the demanding conditions for integration and use of new technology, especially in cases such as WSANs.

**Figure 4:**
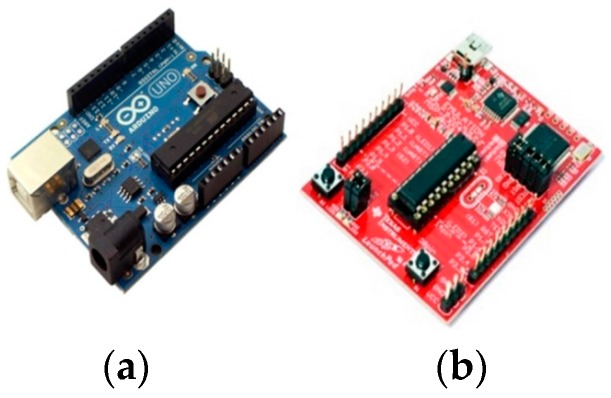
Expandable open-source platforms: (**a**) Arduino Uno; (**b**) Launchpad MSP430.

Today, there are two popular competitive open-source platforms, namely the Arduino [60] (Figure 4a) and the Lauchpad (Figure 4b) from Texas Instruments (Dallas, TX, USA) [65]. For each platform, there are several add-on boards aiming to provide application-specific functionality, produced by the original creators or by third parties such as companies or individuals from various users’ communities. Both Arduino and Launchpad provide several alternatives, in terms of processing power, number of input/output pins and peripherals.

In the meantime, all the key-player semiconductor manufacturers decided to launch Arduino-like, or Arduino-compatible, platforms, in order to promote their own new MCUs and microelectronics portfolios. Among these platforms, they are the Nucleo from ST-Microelectronics (Geneva, Switzerland) [66], the FRDM from Freescale/NXP (Eindhoven, The Netherlands) [67], the XPresso from NXP [68], and Blackfin DSP platform from Analog Devices (Analog Devices Inc., Norwood, MA, USA) [69]. Others, such as Infineon (Infineon Technologies AG, Neubiberg, Germany), have launched application-specific Arduino shields [70]. As the acceptance of the open-source expandable platforms (OSEP) increased, the introduction of the single-board-computers (SBCs) extended the capabilities of such platforms, regarding the processing and computational power, the use of open-source operating systems such as Linux, interface of low-cost USB communication modules (WiFi, ZigBee, Bluetooth, GSM modules etc.), and the connectivity with cameras and screen displays, interfacing with audio sources and outputs, etc.. The most of these SBCs are miniature in their physical dimensions (i.e., credit-card sized), low-power and low-cost compared to mini computers. The expandable SBCs allow very easily the development of web-based applications that is very useful for WSAN applications in remote areas (common in agriculture). Some of the SBCs provide hosting of Arduino shields, in order to ensure compatibility with all the already existing application shields. Thus, the result of this compatibility is the reusability of hardware implementations. 

**Figure 5:**
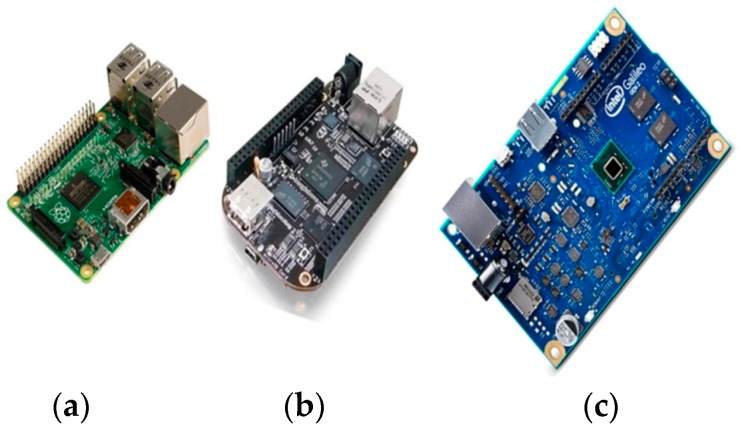
Expandable open-source single-board-computers (SBCs) platforms: (**a**) Raspberry Pi 2; (**b**) BeagleBone Black; (**c**) Intel Galileof Gen2.

Among the most popular SBCs are the BeagleBone (BeagleBoard.org Foundation, Oakland Twp, MI, USA) [71], the Raspberry Pi (Raspberry Pi Foundation, Caldecote, Cambridgeshire, UK) [72], and the Galileo from Intel (Santa Clara, CA, USA) [73]. The most recent of these derivatives, namely the Raspberry Pi 2, the BeagleBone Black, and the Galileo Gen2 are illustrated in Figure 5. Following the introduction of these SBCs, other movements to the same direction took place either from Arduino (e.g., Arduino Tre, Leonardo, and Due) [74], or from well-known semiconductors industries such as Freescale/NXP (FRDM Kinetis KL64) [75]. A well-documented presentation and comparison of all the existing SBCs is given in [76]. In general, the SBCs cannot be considered as a design basis for the build of a WSAN node because of their extended requirement for energy.

The multi-board expandable platform architectures have significantly influenced the typical architecture of WSAN nodes hardware design. The functional blocks that are depicted in Figure 1, is, now, possible to be physically separated from each other thanks to the boards’ mechanical and physical layer “standardization”.

### 2.2. Multi-Board Architectures Expandability Mechanisms

**Figure 6:**
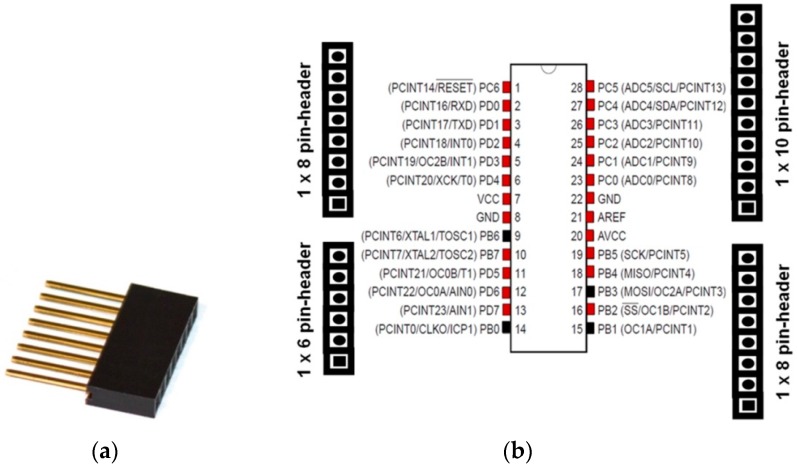
(**a**) An 8-pin boards expansion connector (BEC); (**b**) Arduino Uno Rev. 3 microcontroller’s pin and its four BECs.

In order to connect two printed-circuit boards (PCBs), it is necessary to use board-to-board connectors. On the other hand, in order to connect more than two boards, a solution that ensures boards to be stackable has to be used. Multi-board platforms, such as Arduino, adopted the pass-through pin-headers (see Figure 6a). There is not an established name for these headers. Sometimes, one refers to them as pin-headers with long pins, or just as Arduino headers. In this work, the term *“boards-expansion-connectors”* (BECs) is proposed. BECs are placed and soldered on boards and they usually have a male and a female side, so as to allow for boards stackability. Initially, Arduino architecture used four BECs in total (Arduino Uno Rev. 3) [77,78], in order to connect, in a somewhat random way, all the pins of its MCU (Figure 6b). Practically, the Arduino BECs provide a mechanical and physical access to the on-board MCU. Whilst this technique seems quite simple, in terms of state-of-the-art microelectronics, it freed engineers by giving them a convenient way to design expandable and reusable hardware. Further, this technique is particularly used in radio communication modules that come with BECs, in order to be placed on different MCU-based boards. This capability allows systems designers to plug in and test several alternatives for wireless communication, using the same MCU-based main-board. 

Besides its easy way of firmware applications development through high level APIs, Arduino owes its popularity in the BECs approach for expandability. Any third-party board, which hosts the four Arduino BECs in the exact physical places, it can be considered as an Arduino expansion shield. This way, designers are free to develop the application shields for their particular applications. 

With regard to the applications in agriculture, the typical architecture of WSAN nodes can take a stackable form, allowing, as much as possible, for facilitating the dramatic technological changes (see Figure 3). 

**Figure 7:**
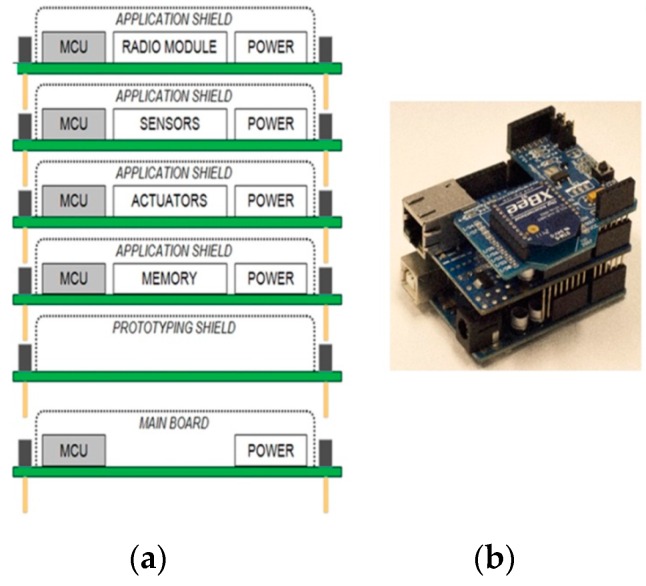
(**a**) The typical WSAN node architecture implementation following the stackable boards fashion; (**b**) an Arduino Uno Rev. 3 expanded with two application shields, one for Ethernet networking and one for wireless communication using an IEEE 802.15.4/ZigBee radio module.

In Figure 7a, an illustration of the physical transformation of a WSAN node keeping all the functional parts of the typical architecture together, but mechanically separated from each other, is given. This approach has started to become popular in WSAN applications development in the agricultural domain and appears to be the solution for the sought reconfigurable WSAN nodes [79,80,81,82]. Figure 7b shows a WSAN node built on one Arduino main-board and two expansion shields, one with Ethernet networking circuitry [83], and a second one with a IEEE 802.15.4/ZigBee radio module (in particular, XBee module [84]). In parallel with the increase of acceptance of the expandable platforms, there has been an expansion of the requirements and expectations to be fulfilled by this approach. Consequently, all the key-player electronics industries have launched boards that keep the mechanical compatibility with the Arduino platform, but they have also put more powerful processing units and extra BECs (even Arduino does so). Of course, the mandated increasing need for systems’ expansion negatively impacts any attempt for mechanical and functional standardization. Others provide development platforms that are mechanically compatible with more than one platforms, e.g., the Arduino and Mbed [66,68], or Arduino and Launchpad [85]. 

In general, there is a race in the industry to provide expandable solutions. In practice, their efforts are focusing on the physical layer design through the introduction of different mechanical expansion mechanisms.

### 2.3. Open-Source-Hardware Architectures versus Open-Architecture Systems

Undoubtedly, OSH architectures have been seen as a significant way to avoid having to design hardware systems from scratch. For WSANs systems, the adoption of the OSH expandable multi-board architectures is very convenient, due to the fact that the developers (engineers and researchers) can test, evaluate and integrate several wireless connectivity solutions coming in the form of plug-in modules together with several MCUs’ main-boards alternatives. This approach increases the degree of freedom and decreases the development cycle time in sake of the applications deployment.

In the following subsections we identify and present several constraints associated with the existing OSH expandable architectures in order to emphasize the need for new solutions that could help the open-source approach to make the next step, namely the step from the prototyping to the optimization and reliability. 

#### 2.3.1. Signals Management Constraints

(a) *Signal conflicts and short-circuits:* According to the expandable multi-board architectures, all the input and output pins (i.e., digital, analog, buses and ports pins) are coming directly from the main-board’s MCU and, through the BECs, are available for use by the rest of the expansion shields. The MCU solely manages every pin regarding its signal direction (i.e., input or output), its function, and its frequency of operation. Expansion shields cannot change the characteristics of the BECs signal-pins. On the other hand, if a pin is declared, for example, as output from the MCU, then, this signal must be an input for the rest of the shields, otherwise serious problems will appear due to electrical short-circuits.
(b) *Limited multi-MCU development:* The egocentric style of pins management by the main-boards does not allow for real multi-processor designs. Thus, it is very difficult to have two or more shields with a MCU in each of them, which, at the same time, are managing some of the signals of the common BECs.
(c) *Waste of existing system’s resources:* In the OSEP-based WSAN hardware implementations it is very common to plug-in a wireless communication module on a MCU-based main-board shield. In this case, the MCU just reads and writes data from/to the wireless module through an embedded serial port or bus (e.g., UART port, I2C-bus, or SPI-bus). In practice, the majority of the wireless modules have their own MCU into which the communication stack is running, while several input and output pins and ports are available to the developer for application use. This means that, in this design, there are two MCUs, but, in practice, just one MCU can be used for the application’s scenario (that of the main-board), so the distribution of processing power among the shields is rather limited and several development resources are left unused.
(d) *Signal voltage level incompatibilities:* Several times, there is incompatibility in terms of the logic levels of the signal pins among the various expansion shields. In the embedded systems circuits, there are two typical logic families, that of +5 Vdc and that of the +3.3 Vdc. In cases where two or more expansion shields, with different voltage logic levels, have to be interconnected through common BECs, then, specific extra logic level translators circuits must be in place. Depending on the direction of the signal pins, i.e., inputs or outputs or both, the voltage level translators should be single-directional or bi-directional. This issue cause extra cost, more physical space on the shields’ boards, and degradation in energy efficiency, as well as reduction of reusability of shields.
(e) *Unused signal pin conditioning:* When the application does not need all of the available pins from the BECs, then, these pins are left floating, in terms of circuit termination. Each particular MCU explicitly defines the signals conditioning for its unused signal pins. The lack of unused pins management can cause significant problems related to the loss of energy, poor electromagnetic noise immunity, low ESD and EMI performance [86], and application scenario intermittent execution caused by erroneous interrupts activation in the MCU’s firmware. The definition of the unused pins level can be done either by enabling the MCU’s internal pull-up resistors or by connecting external pull-up or pull-down resistors. In both cases, there are energy balance disorders. On the other hand, there is no provision for the external resistors in the main-boards or in the rest of the expansion shield boards [87].
(f) *Signal routing inflexibility:* There is no mechanism to terminate the route of the BECs signals at the shields level. For instance, when the MCU outputs a signal to a particular shield, then this signal is needlessly forced to be an input to the rest shields due to the common BECs signal pins. On the other hand, the existing BEC style of mechanical standardization limits the full exploitation of the overall system, since any single signal of the BECs, except from the supply voltages and ground pins as well as the data busses pins, can be used only from one shield and the main-board, so the functionality is sacrificed on the altar of the invaluable expandability and reusability.

#### 2.3.2. Power Management Constraints

(a) *Poor energy conversion efficiency:* Due to the fact tha, all the OSH expandable architecture solutions have originally been designed for pilots and proof of concepts in indoor test environment, they disregard the need for efficient power management. For WSAN applications in the field of agriculture where the hardware nodes have to be battery-operated, the existing OSH architectures entail problems, because these solutions are not energy optimized. Arduino-like as well as the SBC hardware solutions require external +5 Vdc power sources, which in most of the cases is coming from the USB port of a personal computer. Some of these solutions also accept external supply voltages above the +5 Vdc, usually ranging from +7 Vdc up to a maximum of +18 Vdc. To keep the manufacturing cost down, the shields designers’ choice, for the external voltage management, is to use linear voltage regulators. These electronic components don’t require much physical space on the boards, but, on the other hand, they suffer from very low energy efficiency. Also, the higher the external supply voltage value is from the base +5 Vdc value, the more energy is lost in form of heat at the regulator’s package. Therefore, the use of this type of voltage regulators, in the multi-board architectures, downgrades the overall energy efficiency of the final system.
(b) *Inability to ensure the power of the expansion shields:* After the regulation of the external voltage, the voltage supply signal of the MCU, e.g., +5 Vdc, is routed to the related BECs pins in order to power the expansion shields. Unfortunately, the amount of power that can be drawn from the expansion shields is limited by the particular voltage regulator of the main-board shield [88]. When the expansion shields require levels of power higher than that sourced from the main-board, then, some or all of the shields must have their own power source circuits, in order to be able to accept external power.
(c) *Power cabling ataxia:* The high power consuming shields have their own connection terminal blocks or headers separated from the common BECs. In this way, the total shields constructions are suffering, not only from poor energy inefficiency, but also from power cabling ataxia. The power cabling burdens the ESD and EMI performance and makes the system vulnerable to noise interferences. This problem is significantly escalated when, in the multi-board system, there is the need for secondary voltages, e.g., +3.3 Vdc for some shields with low power MCUs.
(d) *No provision for voltage signals other than logic levels:* Various difficulties appear when higher voltages than the basic +5 Vdc and +3.3 Vdc, e.g., +12 Vdc, are required to drive actuators’ loads. In this circumstance, the use of particular external power supply units for the system is mandatory. From the physical layer perspective, none of the existing OSEP mechanisms of expansion is supporting the physical connection of multi-value voltage signals.
(e) *Energizing unnecessary circuits:* In the expandable architectures boards, it is very popular for the main-boards and their shields to have some extra circuitry for general-purpose use, e.g., LEDs, MEMS-based sensors, etc. Actually, this is a very common practice also in WSAN end-solution and COTS solutions [43]. Such extra circuitry may be useful for testing, during the development phase, but it is totally useless in the final in-situ application, because it wastes significant amounts of energy. For battery-operated WSAN systems in the agricultural environment, this testing circuitry degrades the valuable available energy. Unfortunately, the existing OSEP architectures do not have any provision for this constraint, so there is no mechanism for developers to disengage that extra circuitry, in order to build energy optimized systems. To emphasize this problem, a single LED indicator that is blinking inside of a closed plastic box, installed in the agriculture field, is useless for the users and it consumes more energy than that consumed by e.g., a ZigBee RF transceiver.

#### 2.3.3. Firmware Development Constraints

(a) *Lack of code optimization:* The application scenario that is hosted and running in the program memory of the main-board’s MCU, also called as firmware, normally ought to be optimized and tidily developed, so as to ensure the reliability of the ultimate hardware system. In the case of the Arduino-like expandable architectures, the firmware development is mainly implemented into the particular IDE of the hardware vendors. Whilst such IDEs provide many development facilities to the engineers through the use of extensive ready-made APIs or project templates, they produce firmware that is far from being optimized. For instance, the firmware for just toggling a single LED indicator may involve several kilobytes of program code. Every single line of code in the firmware, when it is executed by the processor, consumes a portion of the available system’s energy. In battery-operated WSAN systems, such in the case of remote agricultural applications, wordy firmware is a well-hidden source of energy wastage. Thus, the ease of firmware development is counteracted by the excess in energy consumption. Today, there are programming tools solutions for the open-source developers that can help to the production of efficient and optimized code (e.g., mbed IDE). Hence the key point is the firmware developers to start thinking about the energy effectiveness.
(b) *No provision for multi-MCU development:* According to the existing OSEP architectures, the application scenario of the system is solely developed with the MCU of the main-board. The OSEPs’ IDEs support only one MCU per application. The concept of multi-MCU aspect is totally absent from the design strategies.
(c) *Evanescence of the hardware realm:* The trend of open-source to use ready-made pieces of firmware or even complete projects from the developers’ community is very catty, because the hardware details are totally ignored, or in the best cases, they are partially acknowledged.

#### 2.3.4. Programming and Debugging Constraints

(a) *Peripherals and energy charge*: One of the most convenient and low cost methods to download the firmware to the program memory of a MCU is the in-circuit programming, or else, in-system programming. The hardware implementation of this programming method requires the usage of a certain number of the MCU’s pins (i.e., Vdd, Vss, Reset, SPI pins, UART pins, etc.), which have to be connected accordingly, in order the MCU to be programmed directly by a personal computer via a USB port or by inserting specific external serial programming devices. Whilst, the programming operation takes place once in-house and lasts for a few minutes, the programming circuitry remains permanently on-board. Furthermore, this circuitry may cause electrical conflicts with other shields, because the programming pins are physically routed to the rest of the shields through the BECs, so in most of the cases, it is mandatory to remove any connected shields before a MCU-based shield programming take place.
(b) *Limited debugging capabilities:* Since, the development of the firmware is mostly based on a combination of ready-made open-source parts of code, written by someone else, it is very critical for the system to be able to support real-time debugging with all the shields engaged.
(c) *Lack of support for multi-MCU development:* OSEP expandable architectures cannot support multi-core developments. Practically, the main-board and each one of the expansion shields which incorporate a MCU must host their own programming and debugging circuits on-board.

#### 2.3.5. Robustness and Reliability Constraints

Of course, all the aforementioned constraints can harm, in a lower or higher degree, the robustness and the reliability of the total system, but there are also some additional issues that relate to the real-life applications:

(a) *Lack of compliance to norms and regulations:* Because the majority of the existing OSEP solutions are considered as prototyping development tools, they are not tested and certified in terms of specific norms and regulations for particular application domains.
(b) *Poor system’s integrity and reliability:* The absence of a central power management mechanism, the erroneous electromagnetic sources from sketchy handling of the unused BECs’ pins (these pins may act as antennae), and the uncontrolled performance in the various shields from different vendors, constitute only some of the issues that are responsible for poor reliability. In addition, certain security issues may arise for some MCUs which are very popular in the OSH platforms [89].
(c) *No form factor and encapsulation provision:* Any WSAN application domain has its own particular requirements for the form factor and the encapsulation of the systems in order to facilitate the deployment and to ensure the longevity of the systems. The existing OSEP solutions do not care about the physical dimensions of the final implementation.

## 3. The SensoTube Architecture

Taking into consideration the constraints mentioned in the previous section, the prospect of a new scalable architecture which, on one hand, can maintain the obvious advantages of the OSH expandable architectures, and, on the other hand, can help OSH concept take the next step towards optimization and reliability by provide the mechanisms for avoiding the existing limitations can be reasonably raised. Hence, the OSEP concept can be fully exploited in the WSAN applications even in the demanding domain of agriculture. Therefore, a new architecture, namely the SensoTube, is proposed and described in this section. The grand aim of the SensoTube architecture is to enable a WSAN hardware system to:

(a) *Escape from the structural restrictions of the existing architectures:* The adhesion to the traditional architecturea together with the persistence for miniaturization seems to be rather inappropriate for real-life applications in agriculture. Actually, the size of a WSAN node doesn’t matter [90], and for the case of agriculture, this is evident from the trend to use large-sized OSH platforms.
(b) *Keep the advantages of OSH expandable concept:* The new architecture should maintain the reasons for which the Arduino-like OSH architectures became popular, i.e., the simplicity, the expandability, the reconfigurability and the reusability of hardware.
(c) *Avoid existing OSH architectures’ implementation constraints:* The new architecture should provide new mechanisms, in order to avoid the constraints of existing expandable architectures (see Section 2) and, on the other hand, ensure the highest versatility and flexibility.
(d) *Satisfy all the applications’ stakeholders:* The new architecture should allow designers of different design fields (e.g., power electronics, communications, data acquisition etc.) to easily adapt their contributions. Also, the end-users should have clear and reusable building blocks for their present and future integrations.
(e) *Support the "separation of concern"*: Regarding the research efforts to study new challenging technologies with potential benefits for WSAN systems, there is a trend for decoupling the WSAN from the application [91]. Also, several other studies, e.g., for WSAN nodes’ scenario reconfiguration [92,93], for strategies on WSAN power management [94], for data acquisition development, or for implementing technologies like Wakeup-Radio (WuR) [95], and many others, are indicatory cases where the decoupling from the wireless networking is required. This decoupling is practically achieved either by the addition of more than one MCU on-board, or by the usage of FPGAs, or by the addition of extra RF communication circuits or modules. Such modifications are necessary to overcome the limited boundaries of the traditional architectures, in order to implement the pilots. On the other hand, they may be considered as custom closed-architecture designs. Therefore, the new architecture should ensure the accommodation of research in new and challenging technologies.
(f) *Support modeling*: The new architecture should allow for modeling of the WSAN hardware system. To meet this target the architecture should provide the highest scalability and standardization. In this way, the WSAN systems could be seen from the middleware infrastructure as well-defined functional multi-class objects.
(g) *Ensure optimization:* The systems based on the new architecture should combine the performance level required in real-world WSAN applications [10] with the vagaries of the agricultural domain. Ideally, the systems should have the optimization level of the commercial end-systems, but with the flexibility of a testbed. Furthermore, a provision should be made in terms of the form factor of the WSAN systems and their encapsulation, in order to cover the specific requirements in the open agricultural field. Actually, the name SensoTube reflects the idea of using plastic tubes for the encapsulation of the WSAN systems in the agricultural fields.

The first step towards the foundation of the SensoTube architecture, was to identify every single possible function that should be exist in an ideal hardware WSAN node and to classify the functions into certain groups according to their similarities and their scope. Next, these groups are considered as seven discrete functional layers (see Table 2) by which any WSAN hardware system can be studied, designed and built. As it is reported in [96], any efforts towards the substantial hardware abstraction can increase the fidelity of the characterization and classification of WSAN systems. 

According to SensoTube, each one of the suggested functional layers has to be able to be implemented as a separate expansion shield. In particular, such functional shields have to be:

- *Autonomous:* Each functional layer shield should be fabricated on its own PCB.
- *Dedicated:* Each shield should be designed in order to implement the tasks of the functional layer at which it belongs.
- *Intelligent:* Local intelligence in every shield is necessary, in order to take care of its functions and to allow for reasonable reconfiguration.
- *Uniquely identified:* When a system needs to incorporate several shields of different functional layer, as well as more than one shields of the same functional layer, then each shield should be able to be uniquely identified by the system.
- *Addressable:* The system, according to the execution of its application scenario, should consider the functional layer shields as addressable units.
- *Self-expandable:* In cases where the PCB surface area is not enough to host the necessary circuitry of a particular function, then one or more complementary extra PCBs should be able to be added without, at the same time, to disturb the rest of other functional layer shields.
- *Context aware:* Each functional layer shield should be aware of its environment, that is, to be able to interact with other shields.
- *Testable:* Each functional layer shield should provide plain testing facilities, e.g., connection points for signals testing.
- *Compatible:* Each functional shield should be designed with respect to the homogeneity in form factor and expansion mechanisms.

From the above characteristics, which form the profile of the ideal functional layer implementation, it is evident that the WSAN system should be a multi-processor system. In part, this is a mandatory in several COTS approaches which use MCUs together with MCU-controlled radio modules [97]. On the other hand, the provision of a multi-processor ability will help designers to escape from the egocentrism of the existing multi-board expandable architectures (e.g., Arduino and the like), which whilst theoretically support the concept but in practice only the MCU of the main-board has the total control of the common BECs.

The commercial MCU solutions present in the market today [40], together with the ongoing research on ultra-low power MCUs can guarantee multi-processor operation, even for battery-operated WSAN nodes [98,99]. In particular, among the most significant commercial achievements are the new low-power high performance ARM-based MCUs, which already have found their way into WSAN node implementations [100,101] and the ultra-low power 16-bit MSP430FR MCUs based on non-volatile ferro-electric RAM [102]. At the same time, researchers are striving towards the elimination of MCU leakages [103], the lowering of the MCUs’ operating voltage level [104,105] and the improvement of the internal power management circuits of the MCUs [106,107]. In addition, particular techniques for energy saving in WSAN nodes have already been studied and have shown remarkable results. Some of them have focused on the wakeup and idle states of MCUs [99,108,109], or on the behavior of MCUs as normally-off devices [110,111].

The realization of functional layers in autonomous shields can help the designers to decide which and how many layers are needed to build a particular WSAN system, to work with discrete functional building blocks, to focus on specific systems’ features, to isolate other functional layers from changes at a particular layer, and to work on an add-and-remove basis, in order to adapt to the specific requirements of implementations. 

In order to handle the functional layer shields as autonomous functional blocks, which can occasionally be added and removed from the main system, particular provisions have to be in place, so as to avoid anarchy in the expandability and scalability. All the functional shields have some common characteristics regarding their operation. In particular, they have to share their electrical signals with other shields, they demand either a single or a multi-value voltage source, they have to be in-system programmed and updated, their MCUs must easily communicate with other MCUs from other shields. The proposed SensoTube architecture establishes the necessary mechanism to support these uniformity and openness needs by the introduction of four inter-layer services:

- Signals management
- Communications
- Programming and debugging
- Energy management

**Figure 8:**
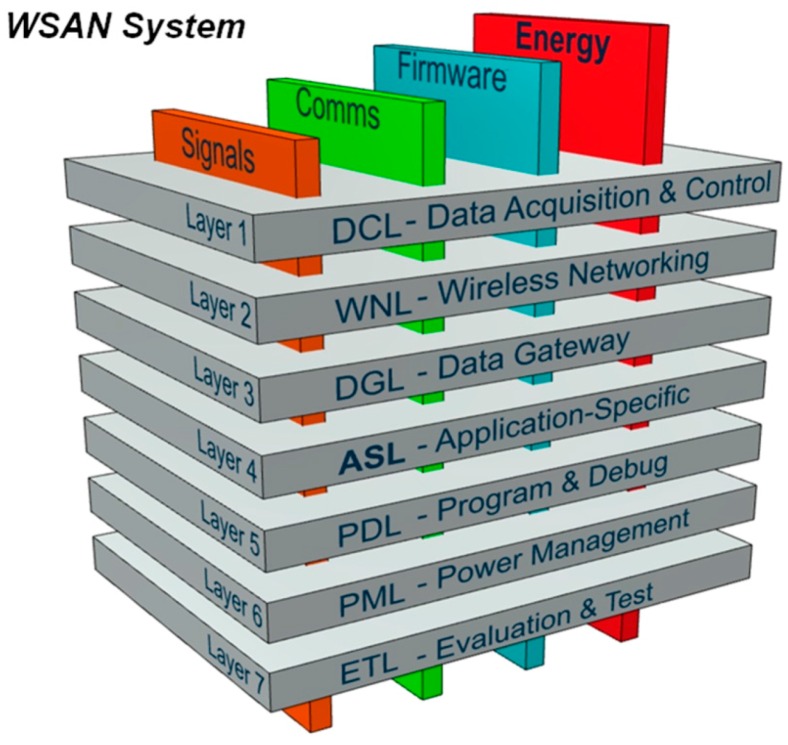
Representation of the SensoTube architecture for WSAN hardware systems design. The seven functional layers are shown as discrete horizontal layers. The four inter-layer services are shown to vertically penetrate the seven functional layers.

Since the four service layers cannot be implemented as distinct plug-in shields, specific provisions have been made in the form of electrical channels in the BECs of the expansion mechanism of the SensoTube architecture, as explained in the implementation reference model in Section 4. Actually, the establishment of the inter-layer services is the key enabler for the realization of the proposed functional abstraction. Without the inter-layer services provision, the elimination of the Arduino-like OSH expandable platforms’ constraints could not be avoided at all. Furthermore, the inter-layer services can ensure the building of a sound, expandable and scalable system. For instance, it is possible to have a system comprised of several OSH main-board shields sharing the very same expansion mechanisms, but at the same time each one of them can be self-expandable and autonomous. A complete representation of the SensoTube architecture is given in Figure 8. At a conceptual level, the presented architecture can satisfy the sub-aims (a) to (g), posed at the beginning of this section.

In the following sub-sections, the usage and benefits of the proposed seven functional layers are described, with particular emphasis on the advantages of the novel mechanisms of the inter-layer services. Additional emphasis is given on the facilitation of challenging WSAN research aspects, and on the solutions to existing design constraints. At the same time, the target is to explain how the WSAN designers can use the SensoTube architecture to adapt their particular requirements. 

### 3.1. Data Acquisition and Control Layer (DCL) 

In real-world WSAN implementations, it is very common for the specifications of the data acquisition (DAQ) and control to change [6]. For example, a new type of sensor may require a higher conversion resolution and a higher sampling rate, or the need for some extra sensors may require extension of the existing analog inputs, or a new actuator may need more energy and special driving circuits in order to be driven, etc. Regarding the field of agriculture, the measuring and monitoring of various physical parameters require, very often, the use of complex sensory devices [5]. For such reasons, in [9] it is pointed out that, regarding the agricultural domain, a data acquisition daughter card is required.

Today, the support of the data acquisition and control aspect of the WSAN systems seems to be rather underrated in the existing architectures. For instance, in COTS (e.g., motes), the emphasis has been entirely put on the RF communications. In particular, some of them have a couple of sensors soldered on-board, just as to be able to demonstrate the networking capabilities in measured data from the wireless nodes [26,43]. In most of the cases, these on-board sensors are not of the proper type and form, in order to be useful in agricultural applications. In addition, as reported in [54], there are serious limitations in data sampling periods, when a single MCU is responsible for both the networking protocol and the DAQ functions under an operating system. In general, the COTS-based systems leave the development of the DAQ and actuators circuitry in the users’ hands. On the other hand, the existing expandable OSH architectures provide limited support for a sound DAQ and control function due to their inherent structural constraints (see Section 2). Furthermore, these solutions are not energy optimized so as to support battery-operated WSAN applications. In particular, the various analog or digital sensors which are connected to an OSH main-board, are always activated, regardless the fact that the sampling rate may be very low. This is particularly evident, for example, in the management of the soil sensors which are based on the SDI-12 bus [112]. Also, the existing expandable OSH-based systems suffer from scalability, in terms of processing power and communication peripherals. Thus these architectures appear to be convenient only for the limited scope of short-term experimentation.

On the contrary, the SensoTube architecture with its inter-layer services facilitates the design and development of flexible and scalable DAQ and control shields. According to the SensoTube, a WSAN system is capable to use more than one DCL shield. Each DCL shield can employ its own MCU. This ensures the ability for reconfiguration at shield’s level, as well as the capability to undertake the execution of measurements scenarios locally without disturbing other functional layer shields. Regarding the MCU, designers can make their choice either by selecting a commercial ultra-low power one, or by using an FPGA [113], or an analog mixed-signal processor [114]. Furthermore, with the introduction of the energy management service, the DCL shield(s) can be entirely powered-ON or OFF, according to the application scenario. In this way, the maximum level of energy consumption control is achieved. Also, a dedicated MCU-enabled shield can help the designers to take all the necessary PCB design precautions for the highest performance in signals integrity (SI) and EMC. Moreover, SensoTube aims to provide the necessary polymorphism, in terms of signal connections, to allow for increased flexibility and versatility. In particular, each of the DCL shields can have its own analog channels and communication interfaces, through the use of the mechanisms of the introduced inter-layer signals management service. For instance, a shield can be self-expanding without disturbing the neighboring functional shields, as well as permit the use of terminal blocks for easy access to signals for connecting external sensors. On the other hand, with SensoTube, there is no limitation of processing, analog channels, and communication interfaces.

### 3.2. Wireless Networking Layer (WNL)

The establishment of a discrete functional shield for the wireless data communication, firstly, allows designers to focus on the wireless networking aspects (e.g., routing protocols [115,116], operating systems [22], new trends [117], new technologies [118,119,120], etc.), and, secondly, supports the requirement for the decoupling of applications from the wireless networking field [91]. 

According to the SensoTube, a WNL can be implemented in its own PCB with a dedicated MCU on-board. In this way, the WNL shield can collaborate with other functional shields for the sake of any particular application scenario. At this shield, any of the known design practices, i.e., chip-set, SoCs, and modules, can be accommodated, in order to implement the wireless data networking. Contrary to the existing architectures, SensoTube allows for the engagement of numerous WNL shields through its inherent expansion mechanism and its inter-layer services provisions. Thus, challenging implementations, such as for heterogeneous communications, as in the case of [31], can be seamlessly facilitated to the WSAN system. Additionally, the specific inter-layer service provision for energy management, allows the WNL shield to be energy aware of every single operation of its data radio communication sub-systems. The ability to have a complete control and monitoring of energy is of crucial importance for the real-world WSAN applications in the field. Also, a WNL shield, thanks to the holistic strategy for the energy management that is achieved by the inter-layer energy management service, can be entirely powered-ON or OFF from other functional layer shields, in order to minimize the energy consumption. On the other hand, the inter-layer service for signals management eliminates the resources constraints of the existing OSH architectures, while, at the same time, provide the polymorphism in expansion mechanisms, in order to help towards the maximum scalability, openness and reusability. This is very important, in the case of the usage of the various integrated communication modules (e.g., Bluetooth, WiFi, and ZigBee modules) in the design of WSAN nodes. According to the existing expandable OSH architectures, such modules are serially interfaced with the main-board’s MCU, in order just to transmit and receive data. In this case, these modules are not fully exploited for the sake of the system. Actually, these modules are built around of a reprogrammable MCU, the signals of which are provided at the module’s miniaturized PCB. A WNL shield can fully exploit the capabilities of these modules. In particular, the analog and digital I/O signals of the modules can be routed to the BECs of the system, and also, through the use of the inter-layer service for programming and debugging, to allow for in-system firmware development. Thus, the SensoTube WNL shields can achieve the integration of such modules in a homogeneous and uniform way.

### 3.3. Data Gateway Layer (DGL)

A WSAN gateway should be able to bridge the local wireless network (e.g., based on ZigBee, etc.) with other communication networks, using proper RF communication modules (e.g., WiFi, GSM/GPRS, GSM 3G/4G, etc.). In contrast to the existing architectures, SensoTube-based WSAN systems can have more than one data gateway channel in the same system through the use of many DGL shields. Thus, SensoTube can effectively support the general domain of the interconnections to external networks [121]. For example, one DGL shield is used for the Internet access while another DGL shield provides Bluetooth connectivity for local user-interface (Human-Machine Interface—HMI), and another DGL shield provides a wired interface via USB or RS-485 data busses for, e.g., local configurations and reporting. The master MCU of the SensoTube-based WSAN system (e.g., the MCU of the DCL, or the MCU of some other layer’s shield) can direct the operations of the WNL and DGL shields through their MCUs, in order to achieve any data interconnection scenario. Furthermore, as in the case of WNL shields, the DGL shields can fully integrate and exploit the inherent capabilities of the modern integrated communication modules (see Section 3.2).

Moreover, the DGL entity can support research and experimentation in the challenging application areas such as the Internet-of-Things (IoT) [33], which at least for now practically appears to be an Internet-of-Gateways (IoG). Similarly, the cyber-physical space (CPS) [122] research field could also be facilitated in SensoTube-based systems. Towards this direction, the processing and memory resources, required for local embedded web servers and other web technologies, can be exclusively designed in DGL shield without disturbing the operations of the other functional layers of the system.

### 3.4. Application-Specific Layer (ASL)

The ASL functional layer can be an application specific shield. This discrete shield can accommodate any functional requirements of the overall WSAN system that does not conceptually fit into other functional layers. On the other hand, the ASL entity can be also considered as a reservation for future needs. In practice, designers could use a MCU-based ASL shield as the director of the rest of functional shields, in order to execute the application scenarios. Such a design option can facilitate the reconfiguration of the system’s intelligence and increase the flexibility in development. Another possible use of this layer’s shield could be the accommodation of various types of memory storage media, in order to store various system measurements, data, operating parameters and execution logs. Such capabilities are very useful in the real-world WSAN applications in agriculture, where the nodes’ data has to be stored locally when the RF network is momentarily down. 

### 3.5. Power Management Layer (PML)

Energy is a very critical factor for the real-world WSAN applications, especially in the agricultural environment, and can influence the lifetime and the reliability of the overall application [18]. As the WSAN technology evolves, the need for power management is increasing [23]. Unfortunately, there are several trade-offs in the commercial WSAN solutions, regarding the use of energy [11]. Traditionally, the WSAN hardware solutions being based either on the traditional architecture and COTS, or on OSH architectures, have been designed without paying particular attention to the energy implications. Furthermore, regarding the provisions for the energy sources, these systems just provide some kind of connection through the use of pins or screw-drive terminal blocks and they leave the users to take care of supplying power, under their own responsibility. In practice, this can jeopardize the overall system’s performance and reliability. 

Additional pressure for energy management is coming from the need to exploit challenging energy-related technologies [34]. The WSAN nodes in agriculture, except from the use of photovoltaic panels [123], can also make use of other, more sophisticated, energy harvesting techniques [124,125,126]. Except for the mature battery types, the harvested energy can be also stored in relatively new media such as the Li-Ion batteries [127], supercapacitors [94,128,129], hybrid ultracapacitors [42], or combinations of thin-film batteries and supercapacitors [130]. The spread of such technologies in real-world WSAN applications entails sound evaluation and modeling. Otherwise they will be limited to pilots and demonstration implementations. In this context, the existence of a separate shield that accommodates all the energy requirements of the overall system is very critical. 

A SensoTube-based system could have more than one PML shield, which can be replaced on an occasional basis, in order to fulfill the scalable energy requirements of the system. With PML shields, the power electronics researchers and designers have a discrete functional shield, into which they can contribute towards the design of energy optimized WSAN systems. At the same time, the PML shields ensure a unified and well-organized energy management that can guarantee the reliability and the lifetime of the WWSAN system. Additionally, with the provision of the SensoTube inter-layer service for energy management, an MCU-based PML shield through the use of on-board electronic switches can power-ON and OFF, in real time, all of the other functional shields. The incorporation of an MCU in the PML shield that will manage the overall systems’ energy could uplift the prospects for optimization.

### 3.6. Program and Debug Layer (PDL)

Programming and debugging are very important functions of a WSAN system [91]. For this reason, SensoTube has made specific provisions. In particular, one or more of the PDL shields can be installed in a SensoTube-based system, so as to accommodate the electronic circuits associated with the programming and debugging functions of the MCUs of the various functional shields. These can be removed from the WSAN system, when the development has been successfully completed and the system is ready for installation in the field. This facility results in energy saving in the final system. Additionally, when there is no support for programming in the abandoned system, it avoids undesirable access to the firmware of the nodes. 

Using the PDL approach also allows one to reduce the cabling complexity in the final system while it permits MUCs of the same technology (e.g., ARM, or MCUs from the same manufacturer), to share the very same PDL shield for their programming and debugging, so designers can provide more compact, energy optimized, and low cost implementations. Another feature, which is very crucial in the cases of remote WSAN applications, is the ability to perform remote upgrades, or else, upgrades over-the-air (OTA) [131]. According to this function, the network stack inside a MCU can be reprogrammed remotely [132]. With PDL shields, this function can be extended also to the remote firmware upgrade of each of the on-board MCUs of the functional shields. Of course, in this case, the PDL should not be removed from the final system. Furthermore, the ability of PDL shields to decouple the programming and debugging circuitry from the MCU-enabled shields, allows for the design of particular circuitry in order to facilitate the connection of novel programming devices that also perform various statistics and energy profiling of the target MCU [133,134].

### 3.7. Evaluation and Testing Layer (ETL)

The behavior of WSAN systems is severely differentiated when they are deployed in the real-world applications environment [21], and practically, this behavior cannot be simulated [135]. Moreover, the detection of possible faults is of crucial importance for the remote system [136]. Traditionally, WSAN designers and developers use various tools for evaluation and diagnostics, referred to as testbeds [29,137]. Ideally, as reported in [135], an evaluation tool should be scalable, flexible, accurate, repeatable, visible, cross-environment valid, and re-usable. Unfortunately, there are very few testbeds available today [138], and, on the other hand, they appear to be inappropriate for in-situ post-deployment testing [117,135]. A thorough study of WSAN testbeds is reported in some studies [139,140]. Through the use of the ETL shields, the SensoTube architecture allows for real-time in-situ monitoring and testing of every single operation of particular circuits and procedures of the WSAN system. In other words, an ETL shield can be considered as the testbed inside the final system. A SensoTube-based system may incorporate more than one ETL shield. An ETL shield is not intrusive on other functional shields and can be easily removed at any time. Among the most interesting testing operations that can be implemented onto an ETL shield are the in-system energy monitoring, the control over the networking protocol execution, the diagnostics of malfunctions in the firmware of MCUs, energy storage monitoring, reliability and lifetime anomalies detection [18,141] etc. Obviously, the ETL entity can open up new horizons for a WSANs’ characterization and modeling based on the systems’ behavior, under real-world deployment conditions.

## 4. The SensoTube Architecture’s Implementation Reference Model

The idea behind the principles of the proposed architecture was to have the WSAN system encapsulated inside a plain plastic tube as those used for irrigation in agriculture. In fact, the very name SensoTube has its roots at this concept. The advantages of this approach are described in depth in Section 6. The definition of a fixed PCB design model is a prerequisite to enable the use of the proposed architecture. The design of the physical expansion mechanism has been accomplished by taking into consideration: the PCB form factor, the fulfillment of the operational requirements of the various functional layers shields, the reusability of the hardware shields, the simplification in modification and cabling, the maximum expandability and openness to support research and development, the easy and low-cost boards fabrication, and the provision of a standardized and uniform way to design the SensoTube-based hardware shields (i.e., to provide a design template).

### 4.1. Printed-Circuit Board (PCB) Model

**Figure 9:**
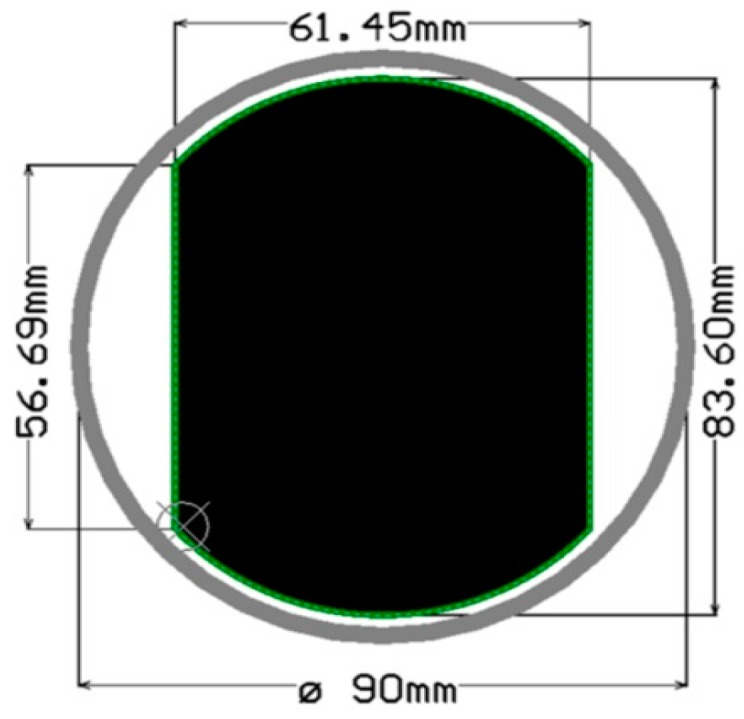
The topographic view of the SensoTube PCB within a 90 mm diameter tube.

The form factor of the SensoTube PCBs is determined from the ability of the boards to be placed inside a tube of 90 mm diameter, as it is illustrated in Figure 9. The diameter of 90 mm allows for enough PCB space. 

Certainly, the spacious PCBs approach is not aligned with the notion of the miniaturization in WSANs design [6] but, in practice, there are no restrictions for the physical dimensions of the PCBs of the WSAN systems in real-life applications in agriculture, where the usage of big waterproof plastic enclosures is a common practice. Because the thickness of the commercially available 90 mm diameter tubes varies from 1.8 mm up to 3.2 mm, the diameter of the board is suggested to be at 83.60 mm. This permits the shield’s PCB to be seamlessly inserted even into the thickest of tubes. The cuts at the right and left sides of the PCB have been intentionally made, in order to reserve enough space for any potential cabling among shields, photovoltaic panel, externally located sensors, and batteries (battery cells should be located at the bottom of the tube and under the shield synthesis). 

### 4.2. Expandability and Inter-Layer Services Mechanisms

In order to support the inter-layer functionality of the SensoTube architecture for signals management, communication, energy management, and firmware programming and debugging, three types of expansion means have been designed and proposed, namely:

- The S-BEC for signals distribution management
- The P-BEC for energy monitoring, control and management
- The J-BEC for programming and debugging of JTAG-enabled MCUs

**Figure 10:**
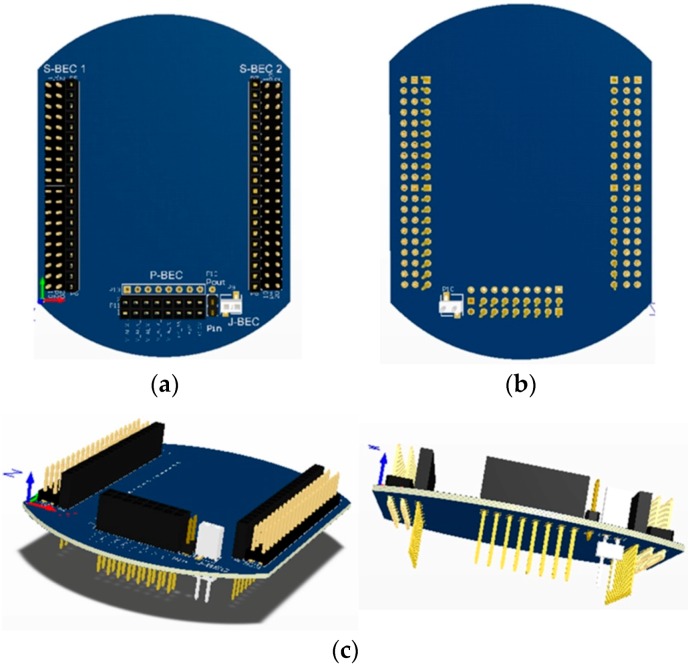
Top (**a**), bottom (**b**), and three-dimensional views (**c**), of the SensoTube PCB model with its physical expansion and stacking BEC-based mechanisms.

These expansion mechanisms have been based on the usage of the popular BECs (i.e., pass-through pin-headers) and they have been enriched with critical technical enhancements. Figure 10 shows the SensoTube PCB model with its three different types of BECs positioned at their exact places. All the blue-colored area is at the disposal of the designer to implement any of the seven functional layers of his system. 

#### 4.2.1 Inter-Layer Signals Management Service Mechanism

**Figure 11:**
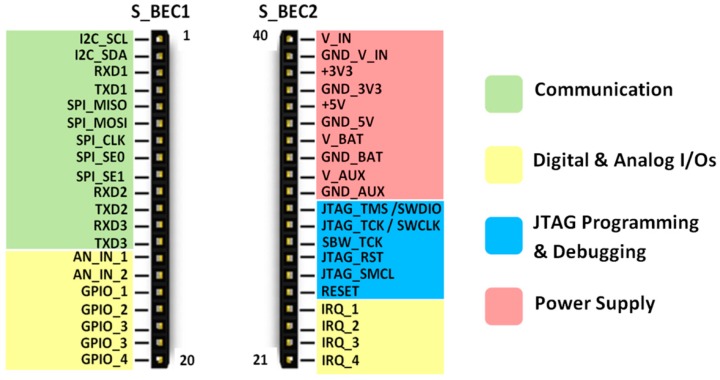
The SensoTube signals BECs (S-BECs) grouped into four functional categories. These pins are physically common among all of the functional expansion shields’ boards.

According to the SensoTube architecture, signals management includes not just the physical connection among the various shields of the system, but also a mechanism for signal isolation and other auxiliary signals connections alternatives. In the proposed PCB reference model two 1 × 20-pins signals BECs, namely S-BEC 1 and S-BEC 2, have been used, as illustrated in Figure 11. The same figure also depicts the proposed types of signals that have been decided to be included in these BECs. As the colors denote, the signals have been conceptually grouped into four functional categories, namely the communication signals (green color), the digital and analog input and output signals (orange color), the signals for programming and debugging through the JTAG standard interfaces (blue color) [106], and the power supply signals (red color). The predefined positioning of the signals on the BECs ensures the standardization for the design of various new shields from conglomerate developers. Forty pins of different types can completely cover the requirements of any WSAN functional shield. Furthermore, the introduction of four exclusive pins to serve interrupt signals can significantly support the design of multi-processor applications, e.g., an MCU-enabled shield can wake up other shields from deep sleep mode, which is a technique, in order to reduce energy consumption. On the other hand, this provision can also support the adoption of challenging embedded systems design techniques, such as event-driven programming, and Synchronous Finite State Machines (SFSM) [142], which can contribute to energy optimization at the system level.

Another novelty is the introduction of ten pins devoted to the power management of the expansion functional shields. In particular, there are five different voltage signals alternatives, two with predefined values, i.e., +3.3 Vdc and +5 Vdc, and three that can be defined by the developer, i.e., the V_IN, the V_BAT, and the V_AUX, respectively. Each one of these voltage input signals has its own ground pin, which is isolated from the rest of the ground pins. This is very useful for mixed-signal circuits design, because it allows the reduction of electric noise interference. Any connections between different ground signals can be implemented at the PCB of any functional shield. The voltage signals should derive from one, or multiple, shields of PML type.

**Figure 12:**
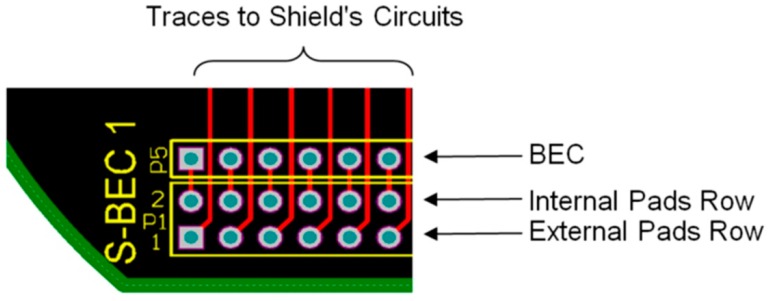
Detail of the S-BEC 1 showing the BEC’s pads row at the top and the two rows of pads added at the bottom. The electrical PCB connections (traces) are denoted with red color.

The two S-BEC signals pins are passing through all the connected functional shields. To overcome the aforementioned signals management constraints that exist in the expandable architectures (see Section 2), SensoTube S-BECs provide polymorphism in terms of the signals connections and routing. In particular, as Figure 12 depicts, two rows of through-hole pads have been added in parallel with the pads of S-BEC 1 and S-BEC 2. The pads of the internal rows are directly connected to the pads of the S-BECs, while the pads of the external rows can be connected to the signals of the shield’s circuits. In this way, the signals coming from the S-BECs are mechanically disconnected from the signals of the shield. 

**Figure 13:**
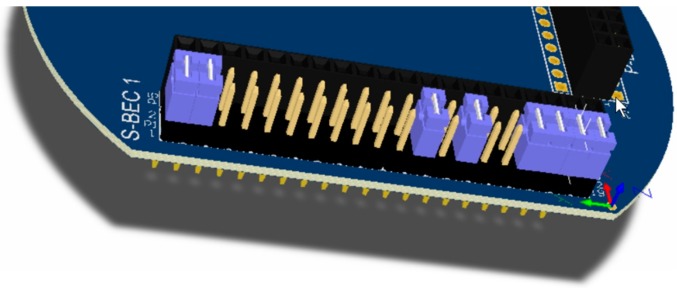
Selective connection of shield’s signals to S-BEC’s signal by the use of pin-headers and short-circuit jumpers.

As illustrated in Figure 13, by placing of dual male pin-headers at the available two rows of pads, and through the use of shorting jumpers, the signals of the shield can be selectively connected to the signals of the S-BECs. This option is very critical for the system’s reconfigurability. Furthermore, in cases where the signals of the S-BECs must be remapped, with regard to the signals of the shield, then instead of the male pin-headers, the developers can make their own wiring at the two rows of pads.

**Figure 14:**
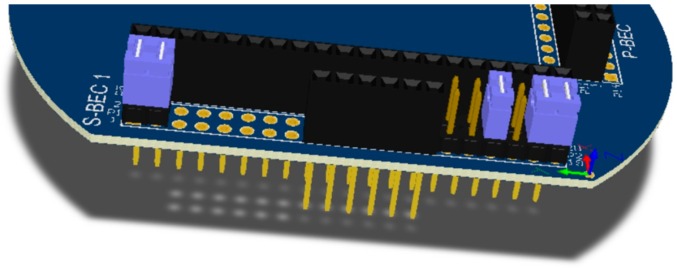
Use of secondary BEC to local connections with neighboring shields.

Additionally, in the external row of through-hole pads, extra BECs can be soldered, in order to enable selected shield’s signals connection to its top and bottom neighboring shields. This is a secondary provision for local signals connections among shields. This can be considered as a nested connection method, and allows a functional shield to have its own sub-functional shields without intervention to the rest of the system’s functional shields. Figure 14 illustrates an example of the combination of a secondary BEC together with pin-headers and jumpers. In this example, just five of the S-BEC’s signals have been connected to the shield’s circuits, while six signals of the shield are ready for connection with its two neighboring shields (or with just one of them). All of these modifications, which are based on the particular usage of the two rows of pads, are not permanent in nature, they do not impose limitations to boards stacking, and they can be performed very easily by the end-users of the shields, e.g., researchers and any kind of developers.

**Figure 15:**
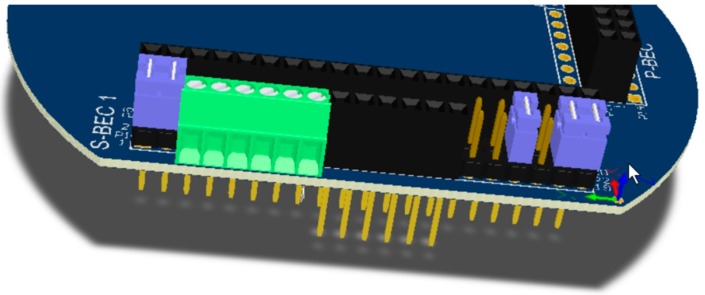
Use of screw-type terminal blocks to facilitate the physical access to the signals of the shield.

Also, there is a third alternative for signals connections, which is very convenient at the system-level signals physical connections. One or more screw terminal blocks can be placed at the pads of the external rows, which are directly connected with the S-BECs signals (Figure 15). This facilitates users’ physical access to the various shields’ signals by just wiring instead of risky soldering. Such, a function is particularly useful for easily adding and removing several types of sensors in WSAN agricultural applications. 

The proposed signal management mechanisms are low-cost and easily implemented in the PCB. The addition of the extra rows of pads (two rows per S-BEC) can be easily hosted in WSAN hardware systems for agricultural applications, due to the fact that, there are no space limitations in this application domain. Despite the fact of the space reservation from the added rows of pads, there is more than enough PCB space for the development of the shield circuitry. 

**Figure 16:**
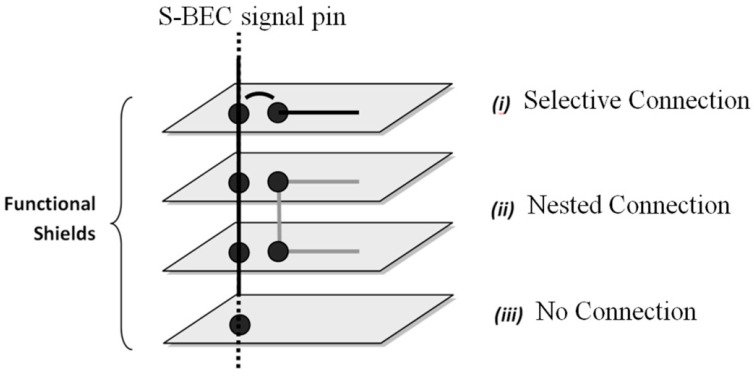
Alternatives in signals connections: (i) selective connection; (ii) nested inter-shield connections; (iii) no connection.

Following the aforementioned signals management mechanisms, the poor performance and low reliability of the WSAN systems, due to clumsy wiring of signals connections, are minimized. On the other hand, the various different shields can be designed as totally independent functional entities without signals connections and boards’ expansion barriers. The SensoTube polymorphism in signals management is depicted in Figure 16.

**Figure 17:**
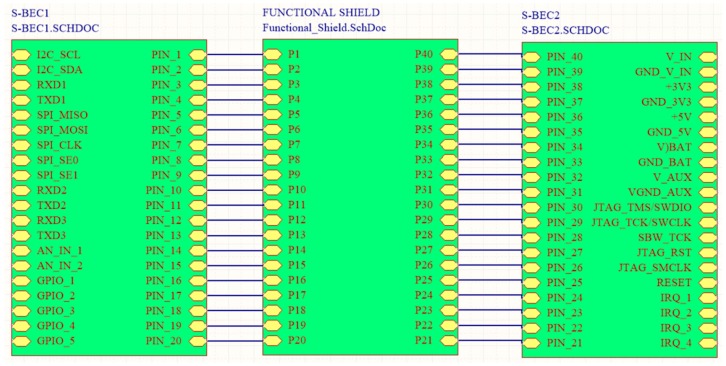
The design template for incorporating the signals management mechanisms of SensoTube into the design of new functional shield. The under design shield is depicted in the middle whereas the standardized S-BECs appear on the left and right respectively.

From the designers’ perspective, the methodology of incorporating the proposed mechanisms is very convenient and straightforward. In Figure 17, on the left and right sides there are the sheet symbols of the S-BEC 1 and S-BEC 2. In each one of these sheet symbols there are the signal ports entities which represent both the common signals of the stacking BECs and the signals of the parallel pads row. The pads row signals are numbered as PIN_1 up to PIN_40. In the middle of the Figure 17 there is a third sheet symbol. This represents the new, under design, functional shield. Designers can choose to connect all, or just some, of the signals of their shield to the pads row’s signals. Signals of similar function e.g., UART transmit and receive signals should be connected to the sheet port entities that are opposite to the TXD and RXD signals of the BECs. There is no physical connection amongst the signals of the shield and the signal of the predefined signals of the BECs. It is on the designers’ discretion to use shorting jumpers (see Figure 13), or secondary BECs (see Figure 14), or screw terminal blocks (see Figure 15) to route the signals of the shield. A detailed usage of the development steps using the SensoTube reference model is presented in Section 7.

**Figure 18:**
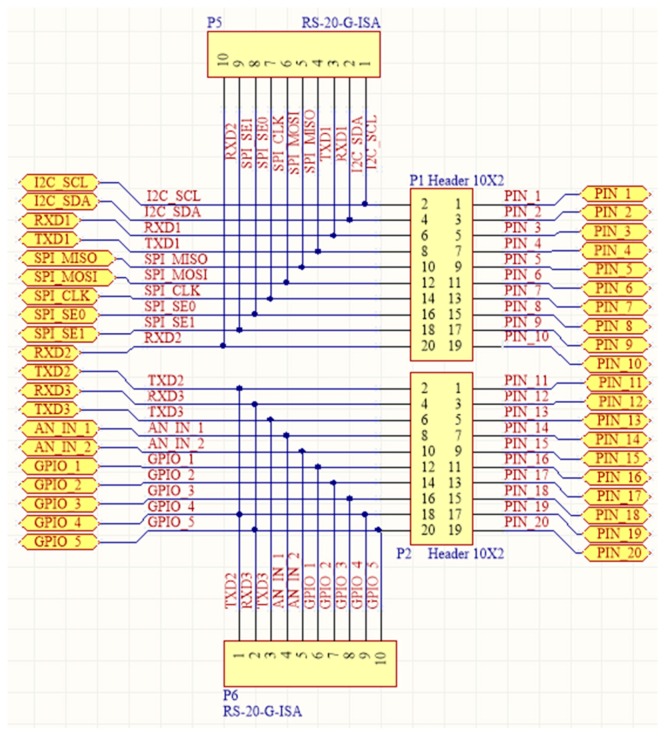
Schematic drawing of the S-BEC 1.

**Figure 19:**
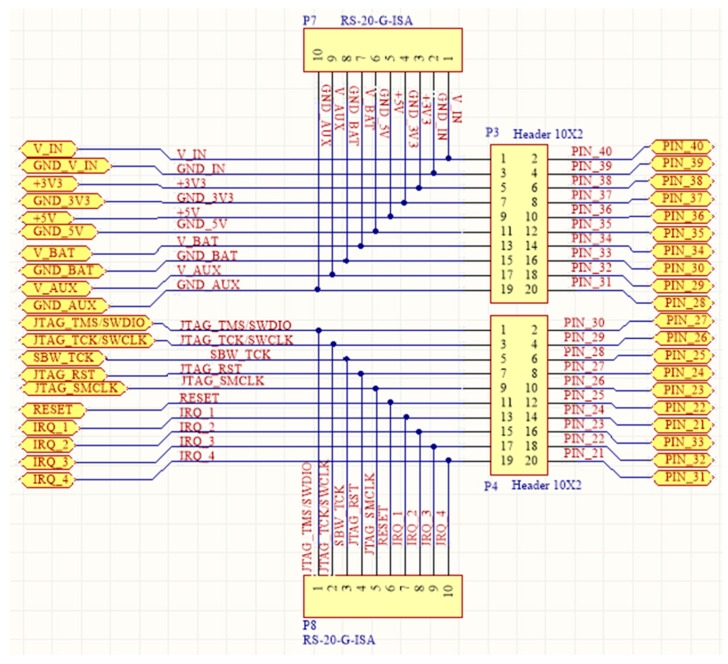
Schematic drawing of the S-BEC 2.

Each one of the above sheet symbols represents a unique schematic drawing file. The use of sheet symbols is a practical and convenient design method for hierarchical structure of a schematic and PCB project that allows designers to re-use ready-made drawings. This facility is common in the majority of the electronic design software suites (e.g., Altium Designer which has been employed in this study). Designers can repeatedly use the schematic sheet symbols and their PCB objects in a copy and paste fashion and put entirely the emphasis on the design of the circuits of the shields. Figure 18 and Figure 19 present the schematic drawings of the S-BEC 1 and S-BEC 2. 

#### 4.2.2. Inter-Layer Communication Service Mechanism

Inter-layer communication includes data and commands transfers among the MCUs of the shields, and among MCUs and various integrated circuits, such as digital sensors, memory chips, analog-to-digital conversion chips, integrated RF modules, etc. In order to support such needs, three types of serial data communication means have been incorporated, namely the I2C-bus, the SPI-bus, and the UART port [143]. The physical access to them can be accomplished through specific connection pins at the S-BECs of the system. From the three, only the I2C-bus can be used for multi-processor communications, because it is a data bus, which can support up to thirty two devices in either a single or multi-master topology [144]. By the use of I2C-bus extenders, the number of supported devices can significantly increased [145]. In addition, the I2C-bus has been proven to be very successful in various applications, as for machine-to-machine interconnection [146]. An additional option for multi-processor communication is the adoption of the CAN-bus, a well-known automotive communication standard [147], which appears to be present in many of the ARM-based MCUs as an integrated peripheral. In this case, some of the general-purpose input/output pins should be reserved for the CAN-bus signals. In the proposed communication signals positions of the S-BEC 1 there are three pairs of UARTs and two chip select signals for SPI in order to avoid resources limitations inherent to the most of the existing expandable platforms. In addition, the SensoTube with the polymorphism in signals connections can ensure unlimited communication resources and, at the same time, it insures the maximum flexibility and openness. 

#### 4.2.3. Inter-Layer Programming and Debugging Service Mechanism

The embedded MCUs at the end-systems can be programmed through the method of the in-system programming (ISP). Basically, the SPI-bus is mainly used for this task together with certain signal, such as the reset, the voltage supply, and the ground signals of the devices that are to be programmed. 

SensoTube supports the ISP method by providing all of the SPI-bus signals to its S-BEC 1 expansion pins, i.e., the MOSI, MISO, CLK and SEL pins. Several MCUs of the interconnected functional shields can be programmed via the very same ISP circuits and by addressing them using some of the general purpose input/output pins of the S-BECs.

Regarding the debugging function, the majority of the in-system emulation and code tracing is traditionally accomplished using specific external devices, which support the IEEE 1149.1 standard for boundary scans, and are widely known as the JTAG debuggers [106,148]. All the necessary signals for the JTAG have been provided at the S-BEC 2 (see Figure 11). The JTAG debuggers are also used for the programming task. From the side of the end-system, a sizable JTAG connector of ten up to twenty pins must be permanently soldered in the PCB. Despite the fact, that there is no use of all of the signal pins of a JTAG connector, its soldering to the end-system is mandatory, in order to ensure the connection compatibility with the JTAG devices. To overcome this design limitation, in the SensoTube, the JTAG signal pins can be routed to a particular PDL shield, on which there is the necessary JTAG connector. The PDL shield could be removed from the final WSAN system, when the latter is ready for installation in the field. 

**Figure 20:**
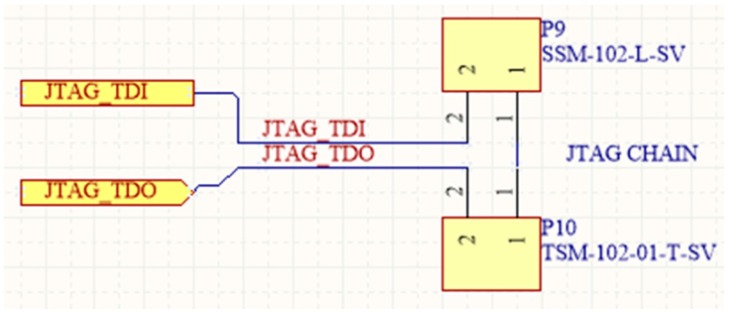
The J-BEC mechanism for the JTAG TDI and TDO data signals’ chain.

In case of the ARM-based MCUs, which are becoming more and more popular in embedded systems [149], the IEEE 1149.1 boundary scan standard can support the debugging of two or more cores, simultaneously. Therefore, through a single interface, the designers are able to perform synchronized debugging of multi-core systems. The multiple cores can be either identical (symmetric multi-core processing—SMP), or different (asymmetric multi-core processing—AMP). The only drawback in the multi-core debugging is the need to use two, instead of one, JTAG connectors at the end systems, in order to implement the necessary scan chain. In particular, the JTAG data output signal from a target system (TDO signal) must be the JTAG data input signal to the next target system (TDI signal) in the chain. To enable the multi-core debugging, the SensoTube reference model has been enriched with the J-BEC expansion mechanism, with which the MCUs of the functional shields can have their JTAG_TDI and JTAG_TDO daisy-chained (see Figure 20). More specifically, two dual-pin connectors have been incorporated for this goal. Since the through-hole BECs cannot be daisy-chained, two dual-pin surface-mount connectors have been employed, one soldered at the top side (the white connector shown at Figure 10) and the other at the bottom side of the PCB. The signal connections, between the two connectors, can be achieved by PCB metal-plated through-holes (known as signal vias). Every SensoTube functional shield should have its own J-BEC connectors soldered onto its PCB. In cases where this feature is not used in a shield, then, the JTAG_TDI and JTAG_TDO signal should be shorted, in order to allow the signals to pass through the neighboring shields. 

**Figure 21:**
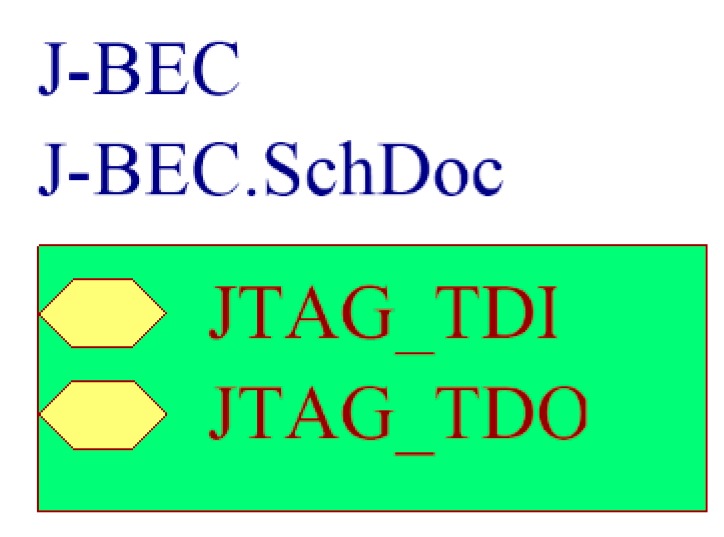
The J-BEC sheet symbol used as a design template for the incorporation of the J-BEC expansion mechanism in new shields designs.

The J-BEC mechanism can be incorporated by designers by simply use the sheet symbol depicted in Figure 21 which represents the j-BEC circuit.

#### 4.2.4. Inter-Layer Energy Management Service Mechanism

The power of the shields has been designed so as to ensure the maximum versatility in development and experimentation. In particular, there are ten independent connection pins at the S-BEC 2 (see Figure 11), which provide all of the functional shields with the necessary voltage sources. The maximum current rating per BEC’s pin is around 5 A and it is more than enough for the vast majority of the WSAN systems in agriculture or other similar application domains (e.g., forestry, environmental monitoring, etc.). 

**Figure 22:**
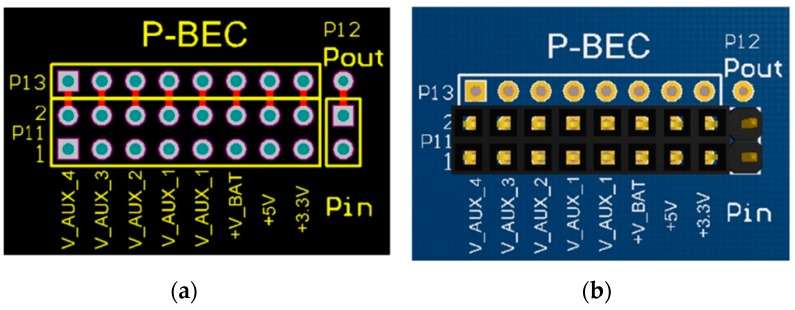
The P-BEC mechanism for inter-layer power management services: (**a**) the schematic drawinng; and (**b**) the three-dimensional view.

In addition to the above typical power supply mechanism, an additional mechanism has been introduced, referred to as the P-BEC. The P-BEC has been designed so as to allow the functions of in-system monitoring and control of the energy in a WSAN node. The implementation of the P-BEC incorporates a 2 × 8 pins BEC. As shown in Figure 22a, the upper pins of the BEC are routed to a row of through-hole pads. The lower pins of the BEC have to be terminated at a PML shield. To ensure standardization in design, certain voltage names have been given to the lower eight pins of the BEC, i.e., +3.3 Vdc, +5 Vdc, V_BAT, and V_AUX0 up to V_AUX4. 

One or more of these voltages can be connected to the upper pins of the BEC by another shield, e.g., an ETL shield, which can monitor and control the energy flow to other shields. Next, with the use of a dual-pin-header (P12 in Figure 22b), any shield can select one of the available voltage sources from the BEC. The pins of this pin-header can either be shorted by the use of jumper, in order to directly provide the voltage source to the circuits of the shield, or help the engagement of various circuits for current monitoring and/or circuits for switching ON and OFF the energy flow. Although, the feature of breaking the voltage supply signals path in order to measure the flow of currents is known in the embedded systems design area [150], the integration of this technique into the WSAN systems is new. 

**Figure 23:**
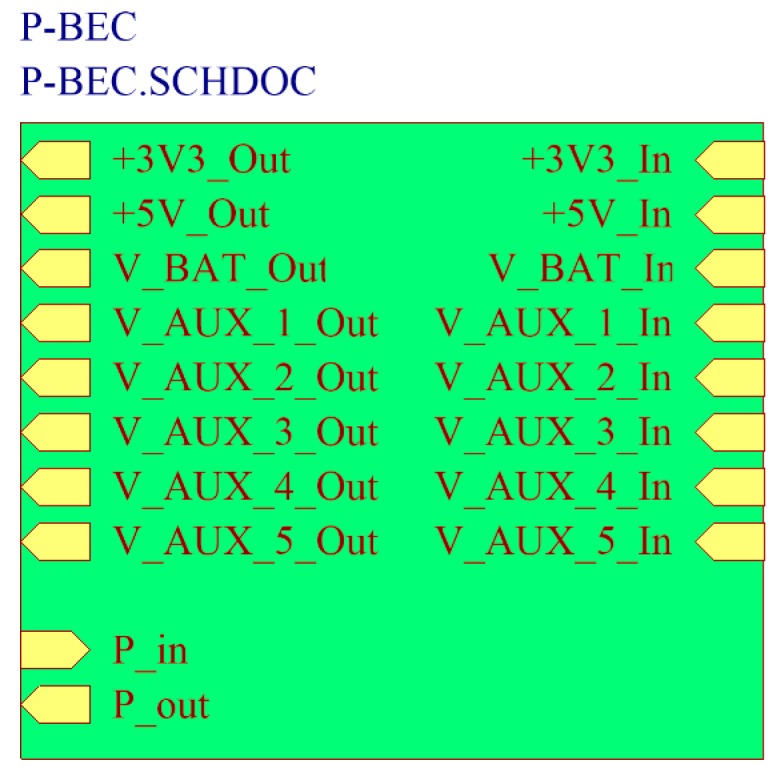
Selective connection of shield’s signals to S-BEC’s signal by the use of pin-headers and short-circuit jumpers.

**Figure 24:**
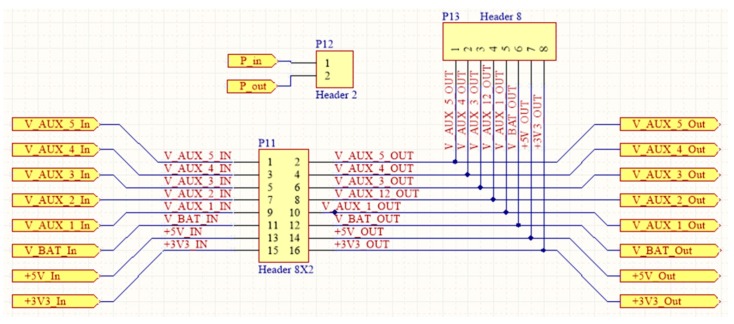
Selective connection of shield’s signals to S-BEC’s signal by the use of pin-headers and short-circuit jumpers.

Figure 23 illustrates the sheet symbol of the schematic drawing of the P-BEC mechanism. The complete schematic drawing file is given in Figure 24. Signals with the “_In” post-fix are considered as potential power signals to the shield, whereas signals with the “_Out” post-fix are those that coming from any other shields, e.g., from a PML shield. 

**Figure 25:**
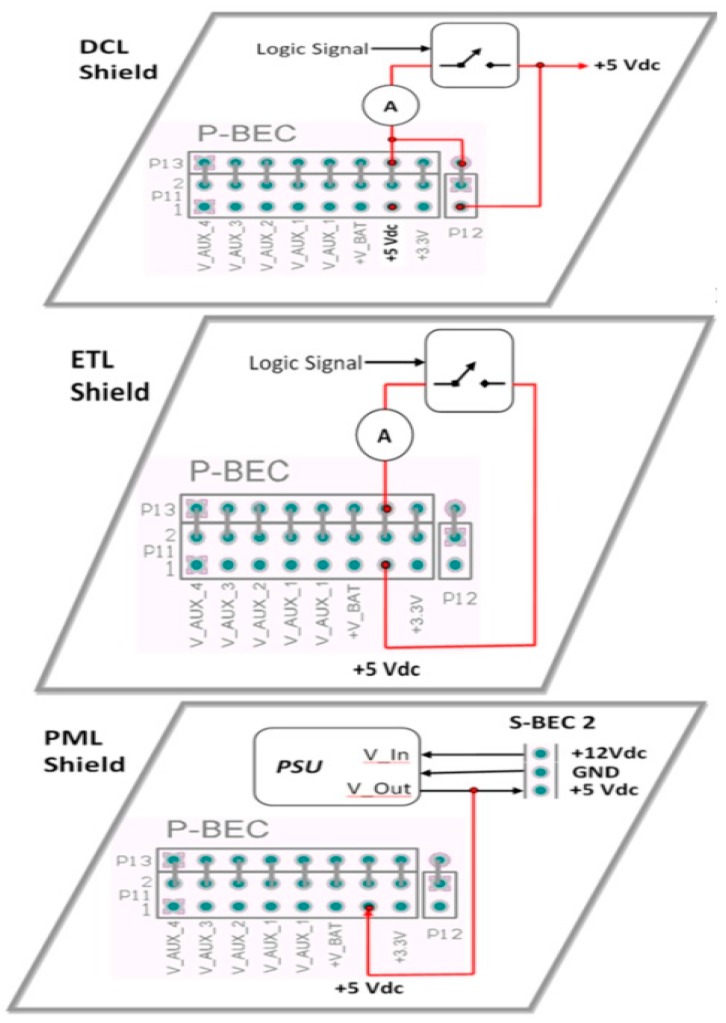
Example of monitoring and control of the energy at group and shield level using the P-BEC mechanism.

Figure 25 illustrates an example of the P-BEC mechanism usage. In particular, a PML shield accepts +12 Vdc and converts it to a +5 Vdc to power any shield that needs +5 Vdc for its operation. The +5 Vdc is then routed to the particular P-BEC’s pin. Through the P-BEC an ETL shield can measure the current flowing to other shields that are using the +5 Vdc for their operation. Additionally, the ETL shield is able to switch-ON or OFF the +5 Vdc voltage source. Such features enable the energy management of a group of functional shields. At the shield level, e.g., in the case of a DCL shield, the +5 Vdc voltage can also be controlled and monitored locally. The logical signals that are mentioned in Figure 25 can be digital output signal of the shields’ on-board MCUs. 

## 5. Support for Firmware, Software and Middleware

As it was explained above, the SensoTube architecture can ensure the development of WSAN firmware applications either in a distributed single-master MCU, or in a multi-master collaborative mode. The proposed particular expansion mechanisms facilitate the use of all the popular development tools, such as programmers and debuggers, for any possible MCU. Moreover, the firmware can be remotely maintained and managed with the use of programming and upgrade over-the-air (OTA) techniques, which can be implemented without intervention on the WSAN system. Regarding the development of the MCUs’ firmware, the designers and the developers are free to use the software tool chains of their choice. 

Except from the firmware, a WSAN system may include the software development of particular PC software applications for either the in-house testing of the system, or for the rapid control prototyping (RCP) (e.g., by the use of Matlab, or LabVIEW software development suites [63]), or for the implementation of the final application for the operation and administration of the system. A SensoTube-based WSAN system completely supports these three tasks by the use of its wireless and wired data interconnection. For the RCP, in particular, any ARM-based SensoTube shield, and through the use of a PDL shield, can enable the hardware-in-the-loop technique of Matlab/Simulink. In other words, the developer can build, execute, and test ARM-based MCUs’ firmware using the Matlab platform. 

In cases of medium up to very large-scale WSAN deployments, the software application development invokes middleware [91]. Towards this direction, several promising methodologies have been reported by the research community. Domain-specific modeling languages based on the Model-driven Engineering (MDE) approach [151] to describe the application, the middleware of systems’ virtualization [152], are only an indication of the current research trends in this WSAN software aspect. In particular, as it is pointed out in [153], it is required for the application and services to be decoupled from the WSAN, i.e., the wireless networking technical operations. In addition, as it is enunciated in [154], the implementation of a substantial middleware would require a layered architecture, through which the overall system could be decomposed into specific modules (layers). Therefore, the abstraction of SensoTube architecture, with the foundation of the seven functional layers appears to be particularly convenient for the development of the middleware. Specifically, the existence of local intelligence in the MCU-based functional shields, together with the inherent support for multi-processor distributed logic, can support the WSAN sub-system’s modeling [155], and allows for the development of comprehensive and substantial libraries of APIs.

## 6. Systems Encapsulation and Installation

In the harsh environment of agricultural domain, the WSAN nodes’ housings, as well as the total mechanical structure of them, is a key factor for the total robustness and reliability of the remote WSAN system [14]. The external influences that a WSAN node may suffer in an agricultural field may include chemical influences (such as acids, etc.), dust, ice, corrosion, air moisture, aggressive constituents of rainwater (such as heavy metals, etc.), solar radiation (UV radiation and high temperature), soil salinity, contamination from birds and insects, contamination from micro-organisms (such as fungi, moss, etc.), and other factors related to air pollution.

**Figure 26:**
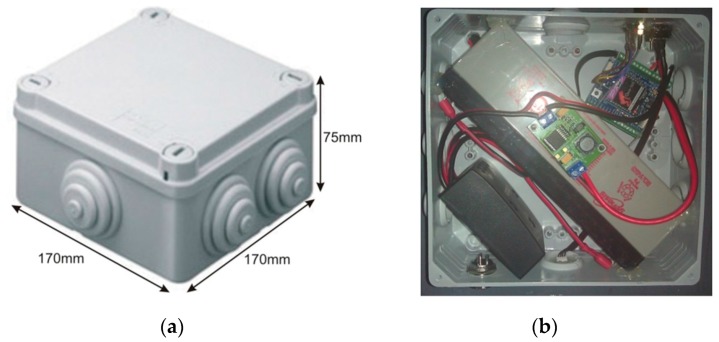
(**a**) A typical electrical IP66-grade enclosure; (**b**) the internal configuration of a WSAN node’s electrical and electronic subsystems.

In order to protect the electronic circuits of the WSAN system from these external influences, particular enclosures proper for electrical and electronics systems are extensively used by both the commercial systems’ vendors and the researchers. These cases are graded according to their resilience to dust and water (IEC IP Codes) [156]. In practice, these enclosures are the only solution for water ingress protection at outdoor deployments, but they are quite expensive and they are not so convenient in terms of the interior configuration of the electronics circuits and other electrical parts, such as batteries etc. Figure 26 shows a typical experimental configuration of a WSAN node for an agricultural application.

**Figure 27:**
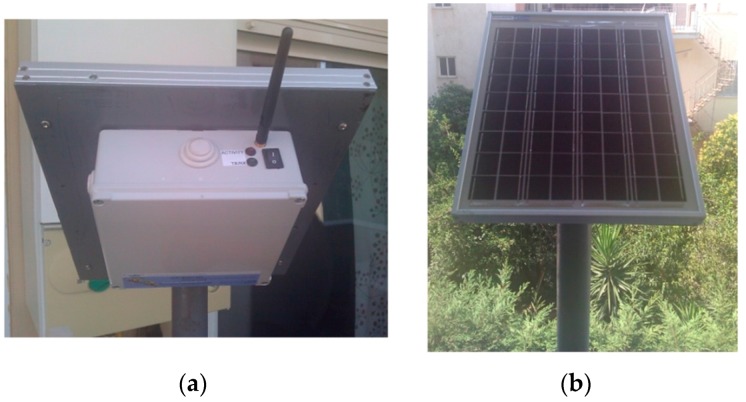
(**a**) An experimental WSAN node’s traditional enclosure installed at the back of a solar panel; (**b**) the front view of a typical WSAN node with its solar panel and its metallic support pole.

In cases where a WSAN node requires a significant amount of energy autonomy, then more than one, or battery cells have to be used on the spot, installed in multiple electrical enclosures at the expense of cost, cabling distribution order, and appearance. Additionally, these enclosures suffer from drilling and cutting, which are frequent functions in experiments, and extra care has to be taken in order to maintain their durability against dust and water. Another issue is the support of the enclosures on the metallic support poles. Figure 27 shows a typical WSAN node implementation with a solar panel, a water-proof electrical enclosure and a metallic pole. In addition, significant complexity is usually added from the RF antennae installation because the antennae must be installed in such places where the electromagnetic signals are not influenced from the metallic materials of the WSAN node. This is the reason why the antennae are typically installed outside the enclosures or, in many cases, at additional support arms. Very often, all these issues are becoming sources for reduced reliability and durability for the deployed system. 

**Figure 28:**
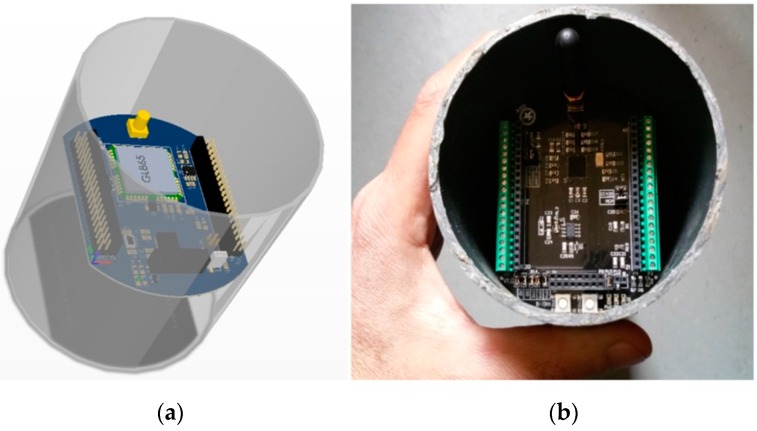
A typical example of the encapsulation of a WSAN functional shield based on the SensoTube architecture. The electronic components of the node are designed in the form of expansion shields able to be inserted within plain irrigation PVC tubes of 90 mm diameter. (**a**) A three-dimensional view showing a SensoTube shield inside a tube; (**b**) a real implementation of the proposed encapsulation method.

Keeping all the aforementioned issues in mind, the use of ordinary drain and water supply tubes are proposed here as an extremely convenient solution for environmental and agricultural WSAN system enclosures. Tubes of polyvinyl chloride (PVC), or unplastisized polyvinyl chloride (PVC-U) inherently provide the required soil and dust ingress protection. According to the proposed SensoTube architecture, the various expansion shields are placed within a plastic tube (Figure 28). The PCB of the SensoTube board model has been designed so as to be fitted within tubes of 90 mm in diameter. The technical specifications of the PVC tubes are shown in Table 3. The selection of the pressure tolerance, e.g., 4 atms or 6 atms, influences the thickness of the tube.

**Figure 29:**
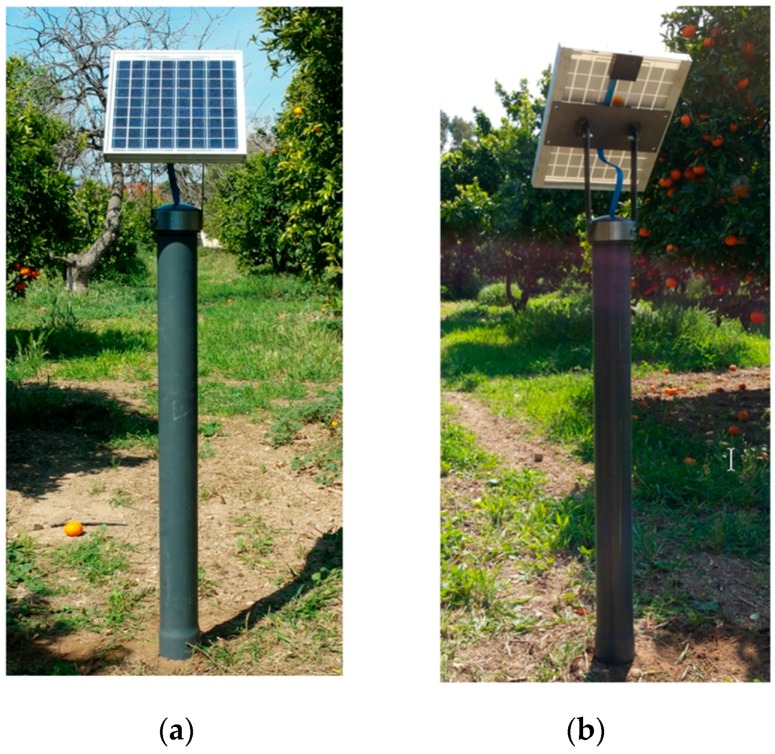
(**a**) A SensoTube-based WSAN node installed in an orchard. All the electronics and the RF antenna of the node have been enclosed within the tube. The solar panel has been adapted at the top cap of the tube (**b**) in such way so as to be easily added or removed.

The very same tube acts also as the installation support pole. The height of the tube may vary according to the precise farming application needs. The WSAN system’s boards can be placed at various heights within the tube. Developers can use tubes of smaller diameters under the boards’ synthesis in order to act as a support spacer. For the battery cells, it is suggested they be placed at the bottom of the tube. This helps the centroid of the tube to be underground. Moreover, the rich set of pipe management accessories, e.g., expansion adaptors, fittings, tees, sleeves, connectors, bends, flanges, etc. can be creatively used for sensory installation above the surface or underground. Figure 29a displays a SensoTube-based WSAN node in an orchard. As it is shown, the plastic tube has been used not only for the encapsulation of the electronic systems but also as a support pole. The solar panel of the node has been easily adapted to the top cap of the plastic tube, as it is shown in Figure 29b. In contrast with the traditional installation methods, such as that of Figure 27, in the proposed installation method the RF antenna is encapsulated within the tube and in this way they are fully protected from the external environment. 

The advantages of the SensoTube approach for the WSAN hardware enclosures are numerous. Some of them are listed below:

- *Ruggedness:* The drain and water PVC tubes by default assure the coveted resistance to water, chemicals, salinity, acids, etc.
- *Non-metallic support poles:* the use of the plastic tube as the support pole benefits the total system because the weight is greatly reduced. Furthermore; the material is inexpensive compared to traditional metallic constructions. In addition, it provides better lightning protection. Finally, it is not attractive to thieves looking for scrap metal.
- *Underground installation:* It is a robust enclosure solution for WSAN nodes in underground installation where the whole of the node, or the most of it, has to be buried underground [158].
- *Internal temperature stability:* The temperature of the air mass inside a PVC tube is slightly different from that of the open air, because this plastic material has a very low thermal conductivity. Also, as deeper the tube is installed underground, the temperature difference is increased due to the facts that a certain part of the enclosure are not directly exposed to the external environment, and that the temperature under the surface is almost constant during the day and night periods. Thus for deployments in environments with high temperature and high sunlight radiation it is possible to keep the temperature of the enclosed electronics at a lower level with regard to the open air temperature. Such a feature is of great importance for the energy balance of the WSAN system.
- *Easy deployment:* PVC tubes are easily transported and, due to their convenient centroid, they do not require deep paddles and cableways for their support on the ground.
- *Larger inner space:* e.g., in a 2.5 m PCV tube of 90 mm diameter and 2.7 mm thickness, the total inner useful volume is about 13,723 cm^3^ whereas the inner volume of a 170 mm × 170 mm × 75 mm electrical enclosure, such as the one displayed in Figure 26a, is just about 2100 cm^3^.
- *RF antennae friendliness:* the RF antennae are installed within the tube and they are fully protected from the threats of the external environment. Additionally, the ability of using all the internal space of a tube facilitates the encapsulation of very long RF antennae, so it is easy to incorporate antennae from λ/4 up to λ (λ is the wavelength of a radio signal, expressed in units of meters). For example, the wavelength of a 433 MHz RF signal is around 69 cm. In this particular case, the ideal antenna should be a 69 cm long wire. In general, antennae close to the wavelength of the incorporated RF signal can benefit the RF signals propagation performance, and they allow for low-cost and low energy antenna driving circuits.
- *Zero RF signal attenuation:* the PVC material doesn’t block the propagation of radio signals. A typical example is the use of PVC-based constructions to hide the antennae of the cellular networks on the terrace of the block of flats at the cities.
- *Neat cabling:* all the cables of the WSAN system can be tidily routed along the inner side of the tube. Hence, the cabling is protected from the environmental influences.
- *Greater energy storage units performance:* The temperature and solar radiation at the bottom part of the tube, which is buried underground, permits batteries and several other alternative energy storage units to maintain their nominal efficiency, capacity and lifetime.
- *Farm machine friendliness:* tubes do not require shoring and cableways for their support. Thus, they allow the unrestricted movement of the various machines and equipment used in farm management.

## 7. Migrating Existing OSH Designs to SensoTube Architecture

The SensoTube architecture allows designers and developers to continue their implementations using the toolchains they already know, and to freely design their own circuits according to their experience and their applications’ specific requirements. SensoTube ensures the above groups of users the necessary expansion mechanisms with which they can successfully make the next step of their open-source designs towards the needed optimization and reliability. 

### 7.1. Towards Energy Optimized MCU-Based Functional Expansion Shields

**Figure 30:**
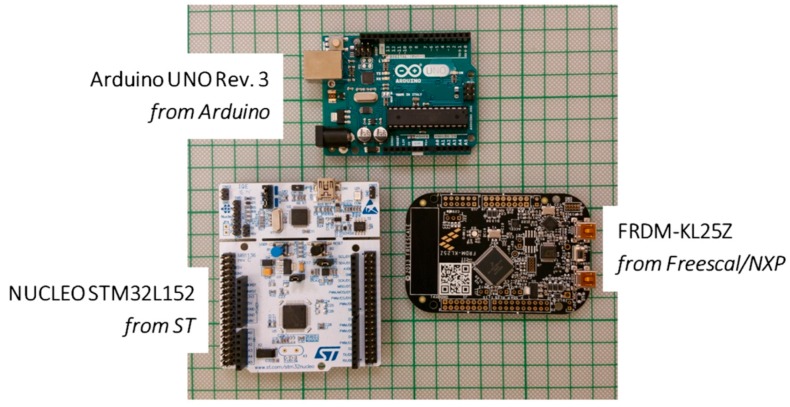
Three of the most popular open-source hardware platforms. Arduino Uno Rev. 3 from Arduino; Nucleo STM32L152 from ST Microelectronics, and FRDM-KL25Z from Freescale/NXP.

As to the question if any one of the existing OSEP main-boards could be used as a MCU-based functional layer shield, or else, if a WSAN system was exclusively based on an existing OSEP main-board, the answer is yes, but the system would suffer from the constraints identified in Section 2 above, and the most important, the system would have very poor energy efficiency. To prove this statement, we chose three of the most popular OSH platforms today, namely the Arduino Uno Rev. 3, the Nucleo STM32L152, and the FRDM-KL25Z. The first one is an 8-bit MCU platform whereas the last two are 32-bit ARM-based MCU platforms (see Figure 30). Our aim was to demonstrate their energy efficiency for a battery-operated WSAN system deployed in the agricultural field.

The methodology of the test was to measure the current drawn by each one of the aforementioned platforms having their MCUs in full active and in deep sleep operation modes. The difference between these two current consumptions is equal to the current required for a functional shield which is using just the MCU circuits and not any kind of auxiliary circuits. The current consumption in MCU deep sleep mode indicates the current consumption of the auxiliary circuits. 

The power supply was decided to be +5 Vdc provided through the USB ports of the three individual platforms. The alternative of providing external voltages greater than +5 Vdc through the external voltage inputs of the boards was rejected because the voltage regulation circuitry of each board is differently implemented and it has different energy efficiency. Therefore, the power supply through the USB ports ensures an equal treatment of the three boards.

**Figure 31:**
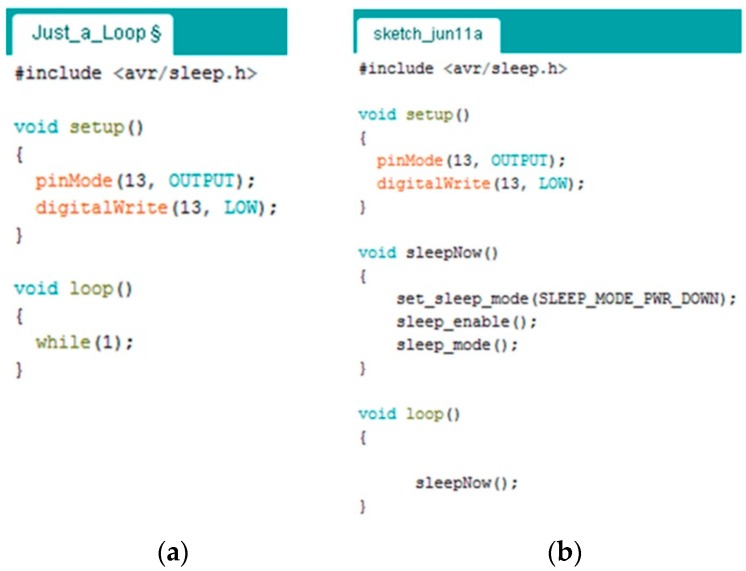
(**a**) Implementation of the full active mode; and, (**b**) implementation of the deep sleep mode. Both implementations have been implemented using the Arduino IDE.

**Figure 32:**
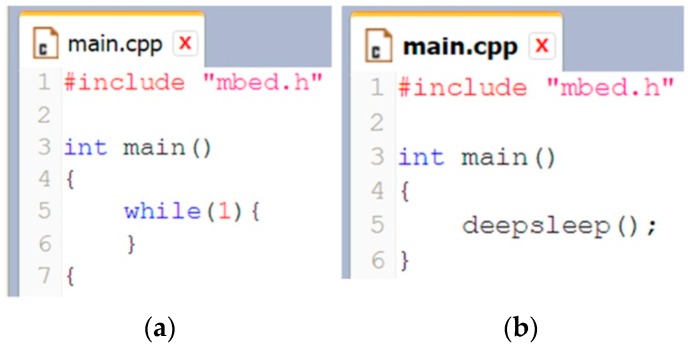
(**a**) Implementation of the full active mode; and, (**b**) implementation of the deep sleep mode. Both implementations have been mae using the Mbed IDE.

The full active state of the MCUs was realized by putting them in an endless loop using a *while* loop programming structure. For the deep sleep mode of MCU state, we used the minimum possible programming functions for each one of the MCUs. The firmware development was easily implemented using the C programming language through two popular integrated development environments (IDEs), namely the Arduino IDE for the Arduino, and the Mbed for the Nucleo and the FRDM respectively. Figure 31 and Figure 32 present the particular codes for the active and deep sleep modes of the MCUs for both IDEs. 

**Figure 33:**
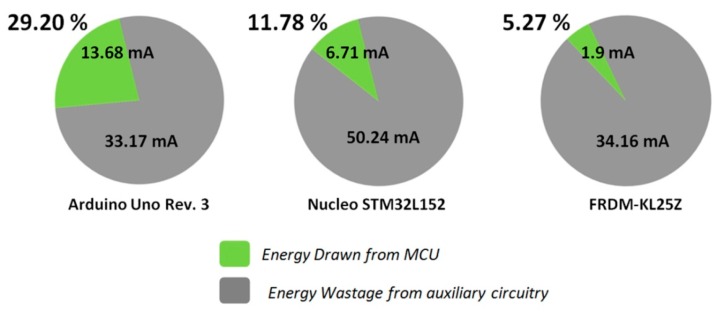
Current consumption of the MCU circuitry (the green parts of pies) and current consumption of the auxiliary circuitry of three different open-source hardware platforms.

The results of the current measurements are presented in Table 4. *I_fa_* stands for full the active current, *I_ds_* stands for the current in deep sleep mode, while *I_mcu_* stands for the maximum current consumption of the MCU circuitry. As revealed, the current consumption due to the operation of various auxiliary circuitry on the boards (e.g., programming circuitry, sensors, LED indicators, etc.) is orders of magnitude greater than that actually required for operating the MCU circuitry. Hence, the use of the existing open-source hardware mainboards is not suitable for battery-operated WSAN systems. On the other hand, following the abstraction concept of SensoTube architecture, more energy efficient WSAN systems can be built. Figure 33 indicates how much energy would be reserved if a MCU-based expansion shield facilitated just the MCU circuitry. 

### 7.2. Development Steps of a Functional Expansion Shield 

**Figure 34:**
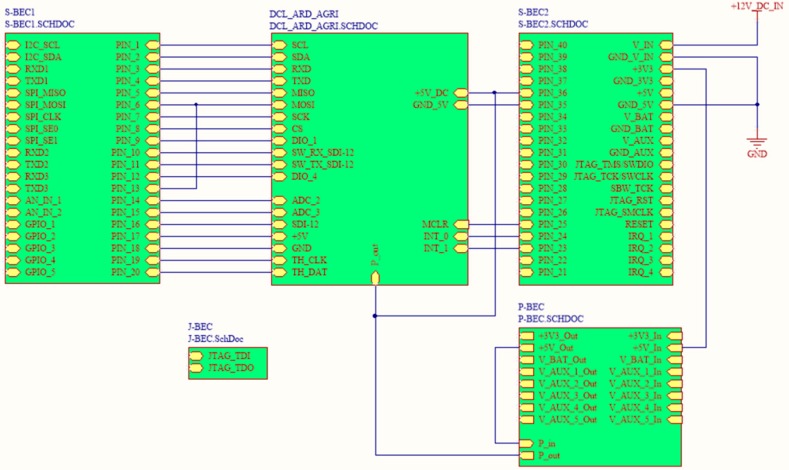
The design of a new shield, e.g., the DCL shield, using the SensoTube design template.

**Figure 35:**
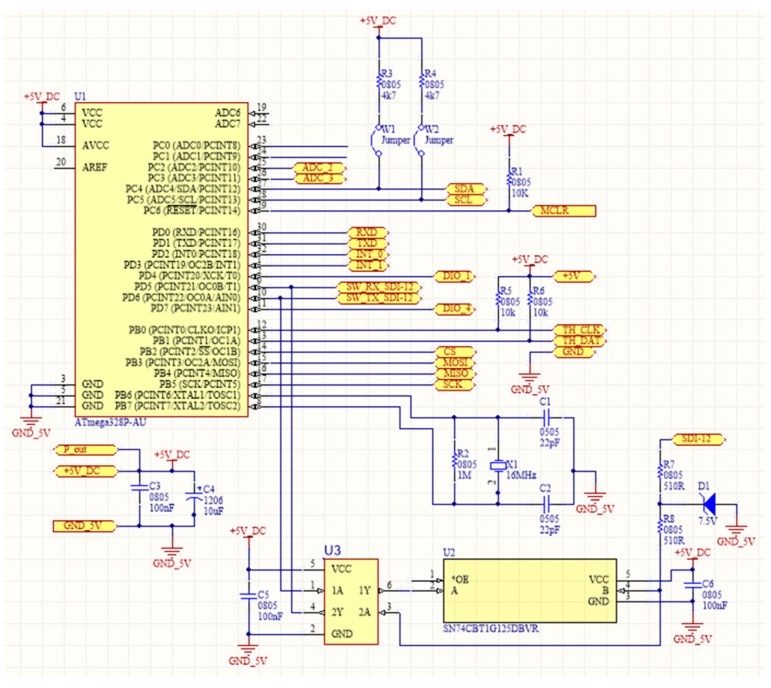
The schematic drawing of a DCL shield’s circuitry, based on an AVR ATmega328 < CU, and supporting the functions of the interfacing to digital temperature/humidity sensors such as the DHT22 and soil moisture sensors compatible with the SDI-12 bus standard.

**Figure 36:**
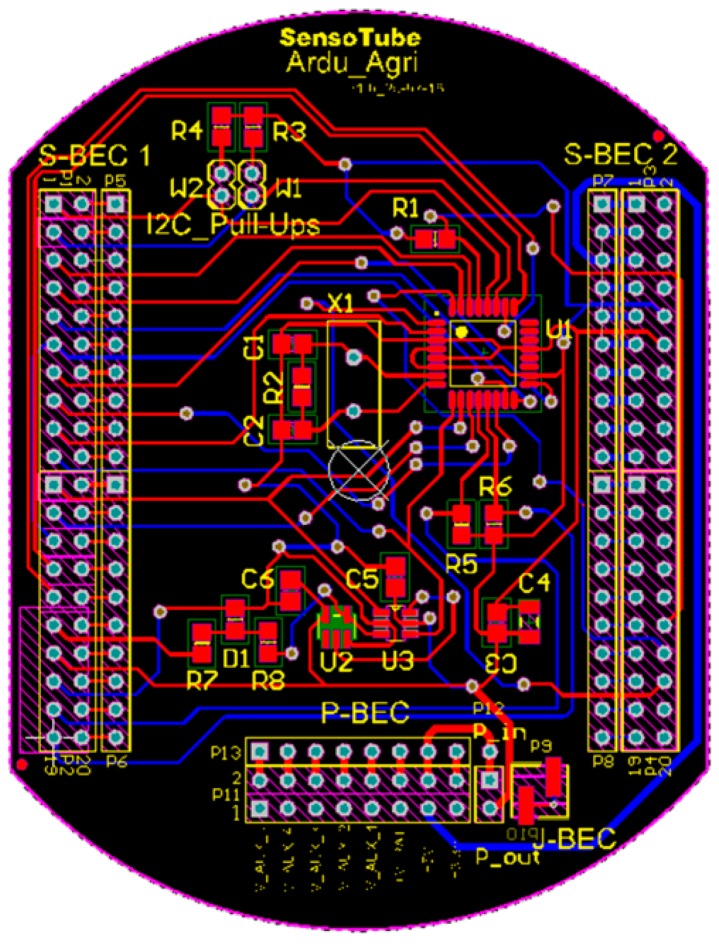
The PCB of the new DCL shield.

**Figure 37:**
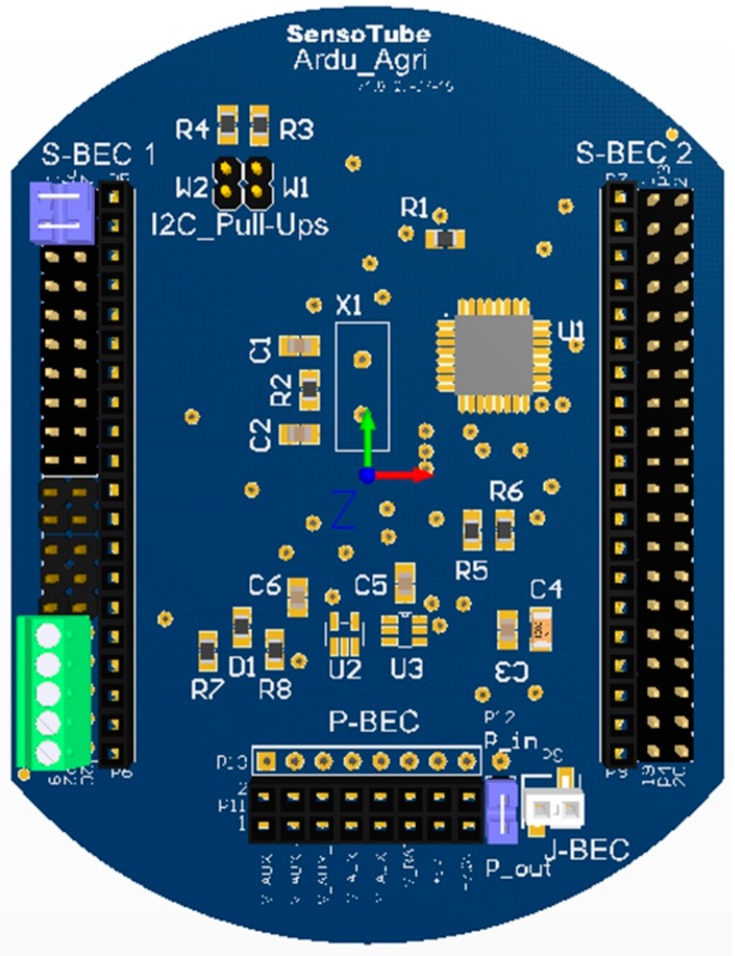
A realistic representation of the fabricated new DCL shield.

As an example of designing a SensoTube-based shield, we describe the considerations and the development steps of a DCL functional shield. This shield will be used for agricultural applications. For this reason, it will have the necessary circuitry for the interfacing with an external air temperature/humidity sensor, and the circuitry for the interfacing with soil moisture sensors using the SDI-12 commercial standard (a 1-wire bus). The specific development steps are:

(1) *Creation of a new design project:* Open a new design project in the electronic design application software tool (EDA tool), adding to this the two sheet symbols and their associated schematic files (see Figure 34). These drawing files contain the connections and parts regarding the implementation of the S-BECs, the P-BEC, and the J-BEC ad they can be used as it is, without making any change or extra work.
(2) *Initiation of the new shield circuitry design:* Create a new sheet symbol for the accommodation of the particular circuits of the DCL shield.
(3) *Consideration and establishment of required signals:* Create the sheet port entities reflecting the particular signal pins requirements of the specific DCL shield. Regarding the temperature/humidity sensor, we have used the popular DHT22 device. This sensor will be installed outside of the system’s enclosure and will interface with the DCL shield through two digital signal pins, namely the clock and the data. This sensor requires a +5 Vdc level power supply. Regarding the soil moisture sensors interface, just one digital signal pin will be required according to the SDI-12 bus. Similarly, thus interface requires +5 Vdc and ground signals from the DCL shield. For the MCU of the shield, we decided to use an AVR Atmega328 due to the fact that it is the basic MCU used by Arduino main-boards. After flashing the MCU with the Arduino bootloader firmware, the MCU will act as an Arduino main-board and the developers can use the Arduino IDE software tool for the application firmware of the shield. As Figure 34 illustrates, on the bottom left side of the DCL sheet symbol, five sheet ports have been added, namely the SDI-12, the TH_CLK (DHT 22 clock), the TH_DATA (DHT 22 data), and +5 Vdc and ground for both interfaces. The rest of the sheet’s ports entities are the remaining available signals of the MCU that can be routed to the S-BEC 1 in order to be exploited in various application scenarios.
(4) *Making the power management decisions:* The next step is to make the necessary connections for the power management of the DCL shield. For the particular shield, we connect +5 Vdc in the P_Out port of the sheet symbol. With this option the shield can be monitored and controlled by other dedicated shields (e.g., ETL shields) in terms of its energy consumption. Alternatively, we create the +5 Vdc and GND_5V sheet ports in order the shield to be able to be powered from the generic power signal pins of the S-BEC 2. The aforementioned ports and connections are illustrated in Figure 34.
(5) *Considerations regarding the programming and debugging of the shield’s MCU:* in our example, for the programming and debugging of the AVR MCU just the UART TX and RX signal pins are required together with the Reset pin of the MCU. All of these signal pins have been added to the sheet symbol of the DCL shield named as RXD, TXD, and MCLR, respectively. Additionally, the SPI signal pins which are also present in the contemplated sheet can be used for the in-system programming of the AVR MCU. The programming and debugging circuitry, as proposed by the SensoTube architecture, ought to be hosted in a PDL shield. The sheet symbol of the J-BEC has intentionally been left unconnected to the DCL shield’s sheet symbol because there is no use of JTAG-based programming and debugging in this shield.
(6) *Design of the schematic drawing of the shield’s circuitry*: the design is achieved following the datasheets of the incorporated components and the signal pins strategy decision made in the previous development steps. In the case of our design example, the DCL schematic drawing is given in Figure 35. In this drawing one can notice the sheet port entities’ names of the sheet symbol.
(7) *Design of the printed-circuit board (PCB) of the shield:* The PCB design must be accomplished with respect to the proposed SensoTube PCB model (see Figure 9 and Figure 10) in order to maintain the standardization of the form factor and encapsulation aspects. The resulting DCL shield is given in Figure 36. The board is a regular double-layer PCB. The connections among the components made manually, without auto-rooting, and it took few hours. In case of auto-routing, the design task could take a few minutes.
(8) *Fabrication and Testing of the Shield:* Figure 37 shows how the new DCL shield will look like after its fabrication. The fabrication of a regular double-sided PCB is very easy, very low-cost and it doesn’t require any pretentious processes. The green-colored screw terminal block placed on the bottom left of the shield (see Figure 37) helps the physical connections with the external temperature/humidity sensor and the SDI-12 bus soil moisture sensors.

The two blue-colored shorting jumpers at the top left side of the board connect the I2C-bus signals of the MCU to the I2C-bus signals of the system’s common BECs in order to allow the intra-shields communication. Additionally, plain dual-pin headers have been soldered to the rest of the pads rows nearby the stacking pass-through headers of the S-BEC 1 and S-BEC 2 in order to selectively connect the general signals of the MCU to the common BECs signals of the system.

### 7.3. Decoupling from the Programming and Debugging Function

**Figure 38:**
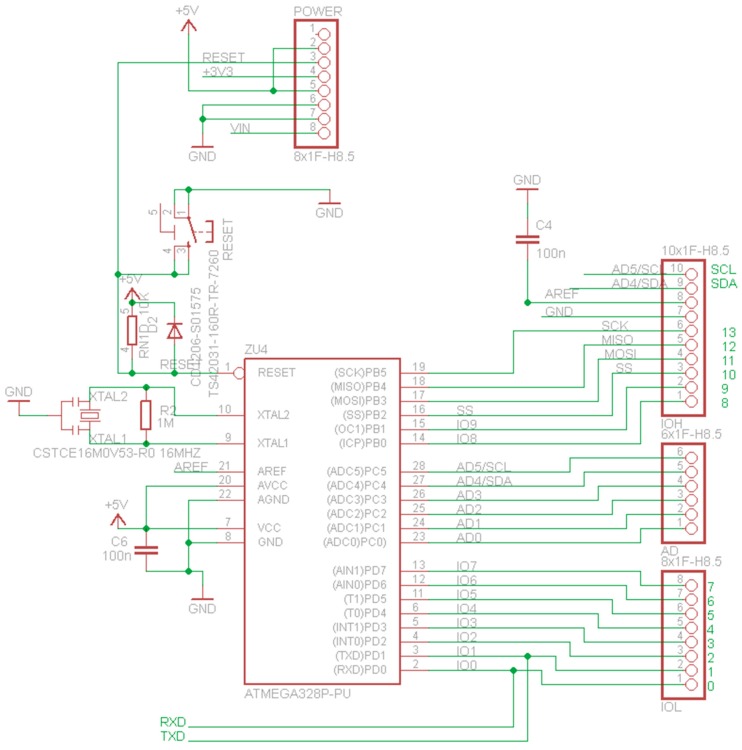
The MCU circuitry of the Arduino Uno Rev. 3.

**Figure 39:**
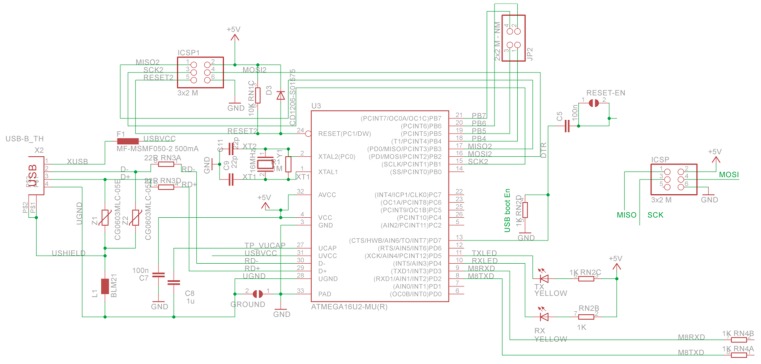
The programming and debugging circuitry of the Arduino Uno Rev. 3.

**Figure 40:**
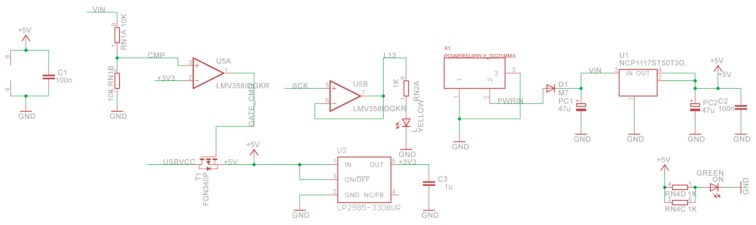
The power management circuitry of Arduino Uno Rev. 3.

The biggest portion of the energy wastage demonstrated in the beginning of this section, is due to the programming and debugging circuitry. According to the SensoTube architecture, this circuitry should be decoupled from the WSAN system through its implementation in the form of a PDL functional shield. As a proof of the proposed concept, we take the case of Arduino Uno Rev. 3 as a use case. We took the schematic file of this specific board from the original webpage of Arduino and we broken ιt down into three parts: the MCU circuitry (Figure 38); the programming and debugging circuitry (Figure 39); and, the power management circuitry (Figure 40). 

The programming and debugging of the MCU of the Arduino Uno Rev. 3 is performed through the UART’s TXD and RXD signal pins (see Figure 38 and Figure 39). The SensoTube facilitates the routing of these two signals through the pins of the S-BEC1. Thus, it is very easy to decouple the circuitries of the MCU and the programming and debugging which can be realized in form of separated functional shields. On the other hand, as it can be seen in Figure 40, the circuitry of power management is devoted just to regulate the external supply voltage and serve power to the MCU circuitry. According to the SensoTube concept, the power management circuitry can be also decoupled from the main-board and to be accommodated onto a PML functional shield. In this way, there is also the opportunity to make such design options so as to satisfy the forecasted energy supply of all of the expansion shields of the system.

Among the most significant advantages of the proposed functions decoupling there are the dramatic reduction of energy consumption at the functional MCU-based shield and the reusability of expansion shields due to their austere implementation. At the same time there are also advantages in terms of the cost of the decoupled implementations. Below we show Table 5, Table 6 and Table 7 with the part-numbers, descriptions and prices of the components used in the aforementioned three Arduino Uno Rev. 3 circuits. The prices are based on unit costs, and they taken from the Mouser Electronics’ webpage (www.mouser.com). 

**Figure 41:**
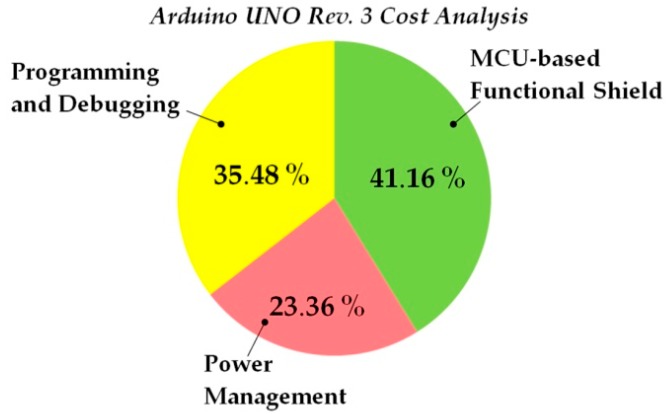
The sub-total costs of the MCU, programming and debugging, and power management circuitries as percentages to the final cost of Arduino Uno Rev. 3.

The sub-total costs from the tables above are analyzed in Figure 41, where it is explicitly revealed that the cost of an Arduino MCU-based shield could be just the 41.16% of the total cost of the Arduino Uno Rev. 3. Thus the proposed decoupling of programming and debugging function from the main-board can result to lower in cost implementations. This is very critical in the case of middle and large-scale WSAN deployments in agriculture where significant cost savings can occur. 

### 7.4. Design and Development Support of Multi-MCU Systems 

The SensoTube architecture provides the maximum possible support for the multi-MCU functional shields as it described in Section 4. In particular:

- It provides a plethora of communication peripheral signals through the S-BECs, e.g., three UARTs, SPI-bus with two distinct enable pins, I2C-bus, etc., which can meet the requirements of many MCU-based expansion shields;
- It provides four interrupt signals to support multi-MCU collaborative application scenarios and functions such as wake up from deep sleep modes;
- It provides BEC signal rerouting or isolation mechanisms at each functional shield in order to avoid signal conflicts;
- It provides a variety of scalable power management configurations;
- It particularly supports 16-bit and 32-bit MCUs, such as MSP430 and ARM cores by reserving specific signal BEC pins for Spy-Bi-Wire (SBW), Serial Wire Debugging (SWD) and JTAG compatible cores; and,
- It supports the programming and debugging of multiple MCU-based shields through flexible, energy efficient, and cost effective combinations.

**Figure 42:**
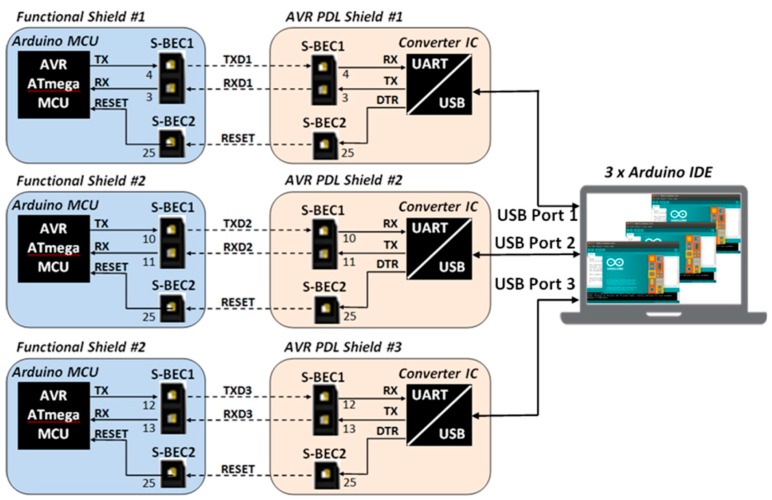
Concurrent firmware development, programming and debugging of three Arduino MCU-based functional shields.

Regarding the programming and debugging of multiple MCUs in the same system we show two use cases. In the first use case we have three functional shields which have an Arduino MCU on-board (namely an AVR MCU from Atmel). To ensure a concurrent firmware development, programming and debugging of the three MCUs we have plug-in three Programming and Debugging Layer (PDL) shields. Each one of these PDL shields has a circuit identical or similar to that illustrated in Figure 39 (programming and debugging circuit of Arduino Uno Rev. 3) and it can be connected via a USB port to a personal computer. In the personal computer there are three instances of the Arduino IDE, one per MCU of the contemplated system. In Figure 42 we show the exact signals connections among the aforementioned expansion shields. Dotted lines indicate the internal signals connections through the shared BECs of the system. In cases where there is no need of concurrent firmware development and testing, then just one PDL shield is enough. Specifically, through the signals rerouting mechanism provided by SensoTube, the RXD and TXD signals pins of the PDL should be rerouted to the associated signals pins of the functional shield. In either case, when the programming and debugging is completed, then the PDL(s) can be totally removed from the system.

**Figure 43:**
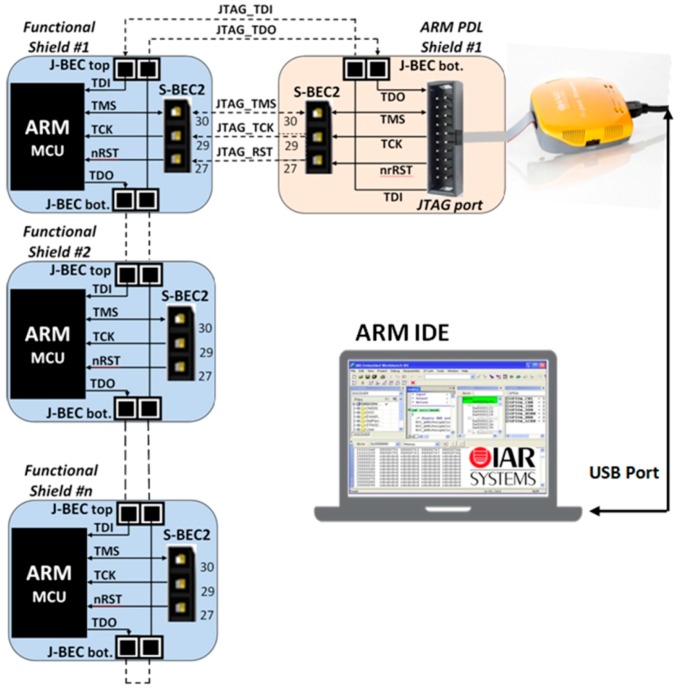
Schematic drawing of the CC430F5137 SoC showing the specific signal names and required circuits for the MCU (MSP430) component of the chip.

A second use case includes several functional shields which have an ARM-core MCU on-boards. In this case, as it is shown in Figure 43, only one PDL shield is required for the programming and the debugging of all the MCUs. 

This is achieved by exploiting the function of the JTAG scan chain. The PDL shield through its JTAG signals pins which occupy in S-BEC2 and in the J-BEC, is connected to JTAG probe (e.g., the I-JET from IAR) and via this probe is interfacing with the personal computer. In the personal computer any one of the popular IDEs can be used for the firmware development. In the use case we chose the Embedded Workbench from IAR. This use case particularly demonstrates the novelty of the J-BEC which actually acts as pass-through BEC but in practice it is comprised by two distinct pin headers (one female on the top side and one male on the bottom side of the board—see Section 4). Of course, the above use cases are just an indication of the real capabilities of SensoTube for multi-MCU support. For instance, a system could comprise any number of different types of MCUs, e.g., Arduino MCUs, ARM-core MCUs, MSP430 MCUs, etc., which through the use of single or several PDL shields could be in-system programmed and debugged. Such a PDL shield which can support AVR, MSP430, and ARM-core MCUs is presented in the following sub-section.

### 7.5. Design and Implementation of a PDL Shield

The aim of this design was to create a single shield which could accommodate, firstly, the various types of JTAG connectors for the most popular MCUs, and secondly the MCUs used in various Arduino main-boards, such as the AVR Atmega328 which is mentioned in the previous sub-sections. Table 8 presents the four different types of JTAG connectors that were decided to incorporate in this PDL shield. Therefore, this PDL could be used to program and debug any ARM, ARM-Cortex, AVR (using Atmel’s JTAG tools), and MSP430 MCUs (from Texas Instruments), which may exist at any of the SensoTube functional layer shields. The first column of the Table 8 gives the specific names of the aforementioned JTAG headers during the design of the PCB of this PDL shield. 

**Figure 44:**
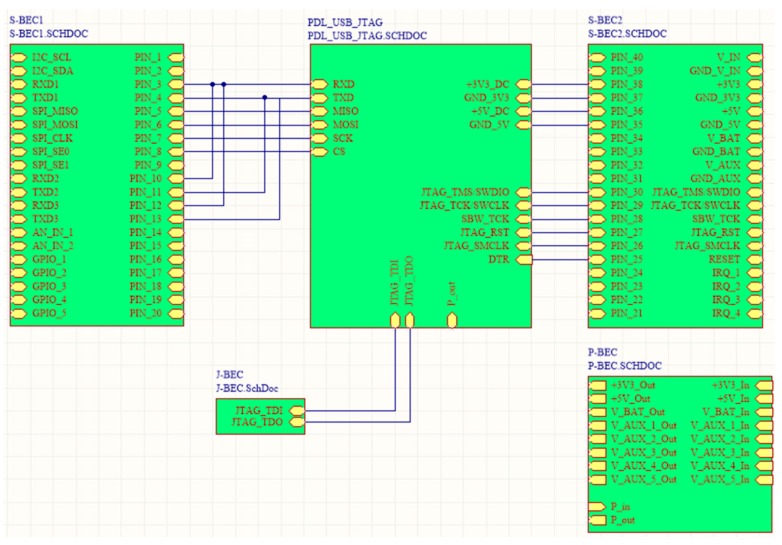
Using the design template of SensoTube towards the design of a new PDL shield with support to Arduino main-boards’ MCUs and JTAG-enable MCUs.

The implementation of the PDL shield was based on the design template which was explained in Section 4 and Section 7.2. The sheet symbol of the circuitry of the new PDL shield is shown in the middle of the Figure 44. As it is depicted, the sheet port entities created in this sheet symbol are relevant to the UART, the SPI and the JTAG signals. Additionally, for extra reconfigurability, the UART TX and RX signals of the shield have been also routed to the other two pairs of UARTs which are present to the stacking BECs of the S-BEC 1. In the case of this PDL there is no power monitoring requirements, that’s why the P-BEC mechanism is not connected to the sheet symbol of the shield’s circuitry. 

**Figure 45:**
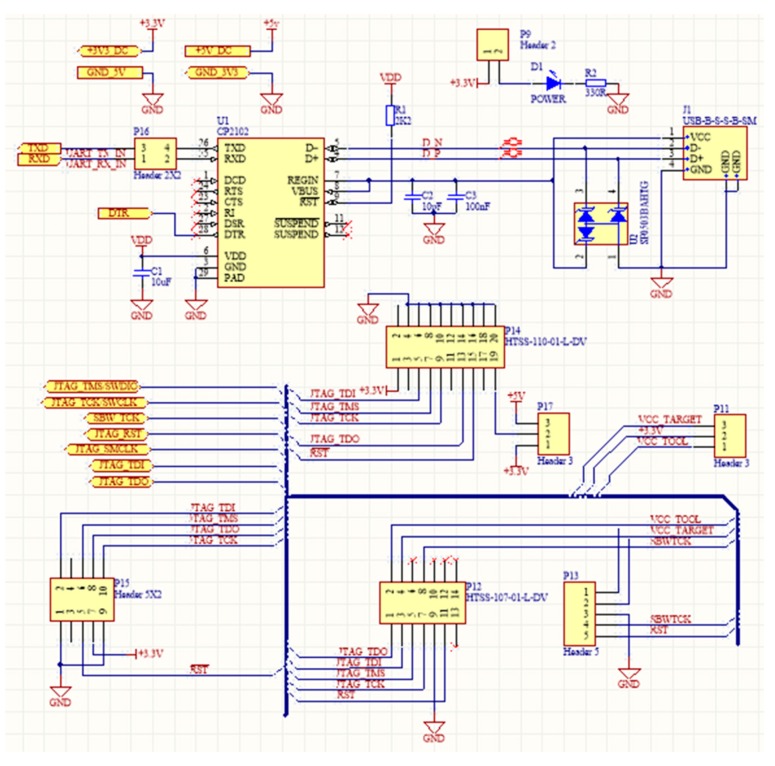
Schematic drawing of the new PDL shield’s circuitry.

The schematic drawing of the shield’s circuitry is given in Figure 45. Obviously, a MCU does not need to be present in this PDL shield. Some extra pin-headers were added, in order to ensure that the target processors could be powered either from the external JTAG devices or from its own system’s voltage source. Furthermore, pin 19 of the JTAG ARM connector was handled in order to be driven either at logic high (+5 Vdc) or low (0 Vdc, i.e., GND). This facilitates the challenging technique of power profiling, or else power debugging, which is supported by the IAR JTAG debuggers [133]. In parallel with the JTAG facilities, this PDL shield could also support the use of other development hardware tools that may use the I2C-bus, or SPI-bus, or even the UART port. The signals of these interfaces already exist in the S-BECs connectors. The circuitry incorporates a UART-to-USB converter chip, i.e., the CP2102 from Silicon Labs, which can be used to program AVR MCUs which they primarily flashed with the Arduino bootloader. Alternatively, this converter can be occasionally connected to one of the three UARTs of the system pins in order to allow a shield to interface with a personal computer. This facility is very convenient during the system’s firmware development processes. 

**Figure 46:**
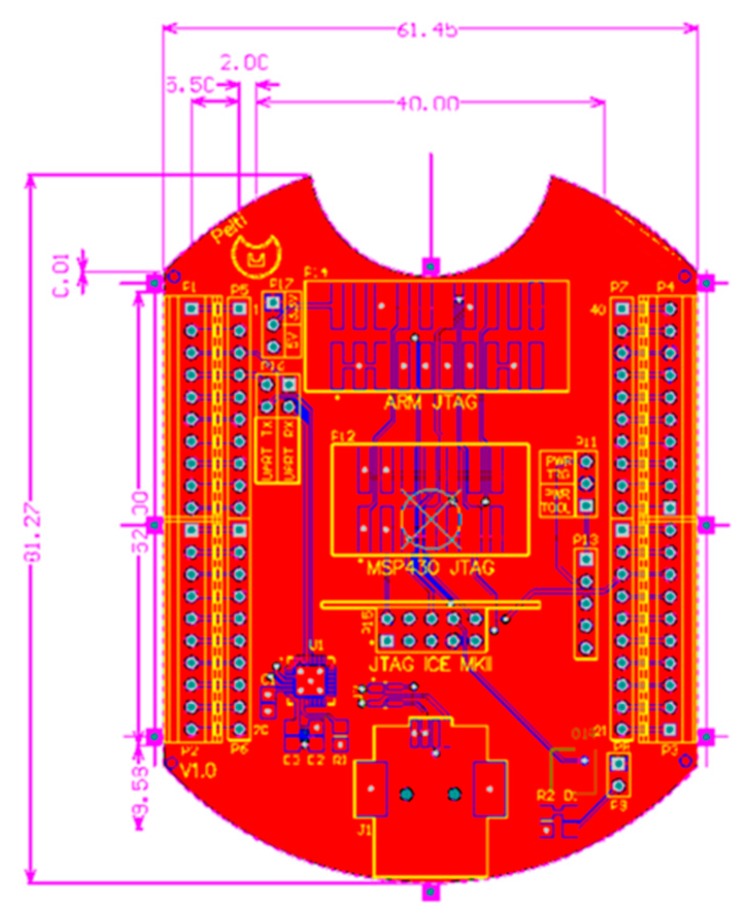
Design of the PCB of the Pelti PDL shield. It is a double-layer board. The red color denotes the top layer and the blue color denotes the bottom layer respectively. At the bottom side of the board there is a USB type B connector for the connection with a personal computer.

The final PCB design of this shield is presented in Figure 46. The semicircular cut at the top side of the board was intentionally made in order to allow any external antenna that may exist in a WNL shield, under the PDL shield. The name *Pelti* was given in this board, inspired by the shape of ancient Greek archers’ shields. The PCB was designed as a double-layer board. All the S-BEC signals have been routed to screw terminal blocks for easy access to them during the development phase. Also, due to the fact that a PDL shield is always placed at the top of the shields’ synthesis in order to allow the easy insertion of the programming and debugging connectors, there is no need to place the P-BEC and the top part of the J-BEC.

**Figure 47:**
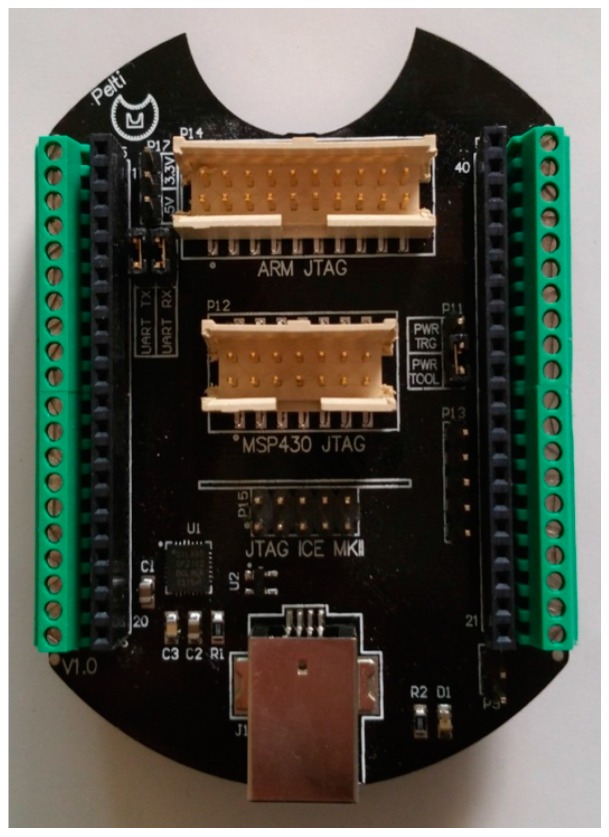
The PDL shield, named as Pelti, with JTAG connectors, USB connector, and screw-type terminal blocks connected to all of the S-BECs signals.

**Figure 48:**
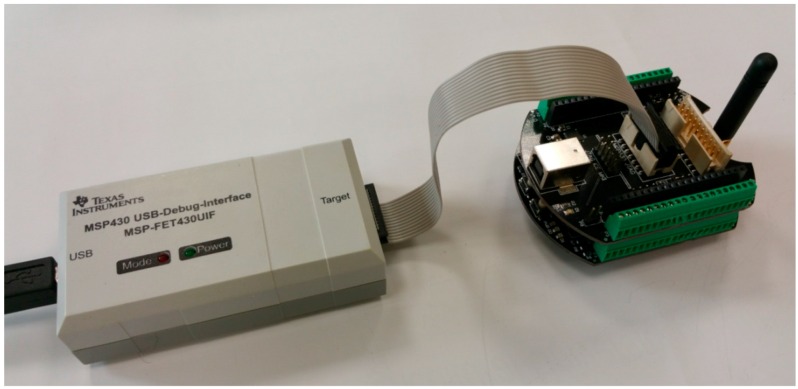
Programming and debugging setup using the MSP-FET430UIF tool from Texas Instruments. The PDL shield is connected onto the WNL shield through the S-BECs and J-BEC for easy access to the required JTAG signal pins.

The Pelti PDL shield, after the manufacture of its PCB and the insertion of its components, is illustrated in Figure 47. Figure 48 shows the PDL shield connected to a WNL shield in order to program and debug the CC430F5137 MCU of the WNL shield through the MSP-FET430UIF tool of Texas Instruments.

## 8. Cost Implications

The SensoTube architecture can significantly influence the cost associated with the most critical aspects of a WSAN application. Both direct and indirect costs can be decreased. The benefits from the reduction of costs can be found in the following three areas of interest:

(1) *Cost reduction at the development phase*: The use of comprehensive functional building blocks, in order to build a WSAN system can reduce the traditionally long development periods, especially in the cases where diverse expertise and skills are required. Thus, the labor cost is decreased and the time-to-market could be shortened too. Towards this direction, the ability of SensoTube to allow designers to use established as well as new innovative tool chains can also help. Moreover, the decomposition of the system into its functions, which are implemented in the form of independent shields, can enable the reduction of the NRE costs associated with the development of the prototype systems because the changes in design can be focused just on particular system’s parts.
(2) *Cost reduction at the systems level:* The increased flexibility, reconfigurability, and reusability ensured by the proposed architecture can positively influence the system’s total cost. In particular, the introduction of centralized provisions for the power supply and the programming and debugging, can release hardware subsystems from integrating superfluous circuits. On the other hand, the ability to add and remove functional building blocks can increase the value of the investment during the lifetime of the application, because any future change can be limited just to the purchase of a single expandable shield and avoid the need to change the whole system. Additionally, the adoption of the plastic PVC tubes for the final encapsulation of the system, instead of special expensive electrical boxes, can help to minimize the cost per WSAN node.
(3) *Cost reduction at the application domain:* A serious problem in the agricultural domain is the theft of the WSAN nodes due to their metallic support poles which can be sold for scrap. This can be prevented by the proposed plastic tubes-based encapsulation, which is simultaneously used as the support pole of the system. Moreover, the proposed approach can significantly help the handling and transportation of the WSAN nodes due to the reduction of the systems weight.

## 9. Discussion and Conclusions

A new architecture for hardware design of WSAN systems was presented in this work. The aims of the SensoTube architecture, as defined in Section 3, have been rather successfully met. The existing end-solutions and COTS-based WSAN systems in their vast majority have been designed based on the traditional architecture (see Section 1, Figure 1), which basically places the emphasis entirely on the wireless networking aspects. This is reasonably explained by the fact that a decade ago the WSAN technology was very new and the efforts had to be diverted towards the design of hardware, which would primarily support the development, the evaluation and the demonstration of that revolutionary technology. Nowadays, when WSANs can be considered as a mature technology and the interest has shifted from the core technology to its embodiment in real-world applications, the rigidity of the traditional architecture appears to constitute a significant barrier for the expansion of WSAN solutions.

The abstraction of the SensoTube architecture provides the necessary design framework with which the real needs of a WSAN system can be accommodated, so each functional aspect of the system can have a clear, predefined space, in order to be tidily implemented without, at the same time, conflicting with other functions of the system. In particular, the seven functional layers proposed are: the data acquisition and control layer (DCL), the wireless networking layer (WNL), the data gateway layer (DGL), the application-specific layer (ASL); the programming and debugging layer (PDL), the power management layer (PML), the evaluation and testing layer (ETL). Thus, the hardware designers, as well as the developers of different specializations, can know in advance how to jump into the WSANs field and at which particular function their design contribution belongs to. Hence, the proposed functional abstraction can support the integration of several challenging technologies that could potentially have a positive impact on the design of real-world application-oriented WSAN systems.

Regarding the practical side of the hardware design, the seven SensoTube functional layers’ ability to be realized in the form of discrete expansion shields ensures that the benefits of the popular open-source hardware platforms will be experienced by all those involved. However, it is totally meaningless to design any of the new functional shields just as another expansion shield for one of the existing expandable platforms, because, although these platforms are very convenient for small-scale prototyping and proof-of-concept implementations, in practice, they cannot be considered as a reliable solution for real-world WSAN applications, such in the case of agriculture (see Section 2). Also, an insurmountable obstacle in the adoption of the existing expandable OSH platforms is that they do not allow for real scalability and expandability, which actually is a key factor for the accommodation of a hardware abstraction, such as the one proposed by SensoTube. In accordance to the architecture of these platforms, the type and the number of the extension shields is determined by the features of the MCU of the main-board, so it is very common for designers to face resource limitations, such as in the processing power, the program memory size, the input/output signals, or the communication interfaces, and the only way to respond is to replace the main-board with one that has enhanced features, but jeopardizing in this way the compatibility with the already existed expansion shields. Moreover, practically none of the existing main-boards support the engagement of MCU-based shields, they do not care about how the expansion shields will fulfill their particular energy needs, and, they do not pay any attention on the form-factor of the expansion shields. Therefore, there are serious risks and troubles in WSAN systems based on these architectures.

In order to overcome the egocentrism of the existing expandable OSH architectures, SensoTube architecture introduces the concept of the inter-layer services. In particular, there are four inter-layer services, according to the SensoTube: the inter-layer signals management service, the inter-layer communication service, the inter-layer programming and debugging service, and the energy management service. These services are easily implemented by the use of BECs and with some enhancements at the PCB design level (see Section 4). With these four straightforward and comprehensive supportive mechanisms, it can be guaranteed that a WSAN system can be built not by using partly suitable expansion shields with superfluous circuitry, but with functional dedicated shields. SensoTube, with its seven functional layers shields, together with the four inter-layer services, can really be used to decompose a WSAN system into its vital functions and ensure the desirable separation of concerns. Furthermore, the introduction of polymorphism in the signal connections together with the anticipation of shields’ local intelligence, by supporting the multi-processor collaboration development, allows for the facilitation of the embedding of any challenging technology into the WSAN system. For instance, it was explained how to exploit the SensoTube architecture in order to host, on an occasional basis, challenging features such as energy monitoring and control, over-the-air programming, and so on. Fortunately, the recent progress in MCU technology allows for several ultra-low energy solutions, and there is practically no change either in the system’s energy balance or in the manufacturing budget. On the contrary, through the use of software and hardware techniques for, e.g., idleness, the multi-processor approach can contribute towards energy consumption reduction. 

Moreover, the SensoTube architecture can essentially support the modeling of the component functions of a WSAN system not relying on simulation and arbitrary approaches but on the basis of the real behavior of the operational entities under the post-deployment real-world conditions. The ability for a realistic modeling together with the energy management insights of the SensoTube-based systems can lead to the design of really energy- and function-optimized WSAN solutions that will meet the demands of applications not only in the agricultural domain, but also in applications such as the forestry domain, the environmental monitoring domain, the wildlife monitoring domain, and others. To further support the real-world deployments in agriculture, particular emphasis was given in the encapsulation of the systems, so plastic irrigation tubes were chosen to be the system encapsulation, because they constitute a novel, low-cost, and extra durable medium that not only can withstand the harsh agricultural environment, but can also allow for better facilitation of antennae, energy storage media, etc. (see Section 6). 

In conclusion, the SensoTube is an open architecture that allows the design of scalable, flexible, reliable, and optimized WSAN systems, while, at the same time, it ensures enhanced versatility by facilitating novel and challenging technologies by multi-skilled designers. As presented through certain use case examples (see Section 7), the migration of existing open-source designs to the SensoTube system entails several advantages for energy efficiency, explicit circuitries’ design, multi-MCU concurrent development support, and low cost.

The cost of implementation has been kept to the minimum, because the proposed novelties have been limited just to the system’s physical layer, i.e., to the PCB design. With the SensoTube architecture, the WSAN stakeholders (see Section 2) can have the maximum expandability, scalability, reconfigurability, reusability, and standardization needed in order to fulfill their particular requirements. Additionally, as it was revealed from the literature review (see Section 5), the SensoTube architecture can be a significant contribution towards the requested hardware abstraction need for the development of sound middleware. 

Future work on the subject includes the design and implementation of an extensive series of functional layer shields and the development of the hardware abstraction layer (HAL) set of APIs with emphasis on the support of middleware requirements.

## Figures and Tables

**Table 1:** Different perspectives of WSAN node in the agricultural domain.

Area of Interest	What a WSAN Node Means
Embedded Electronics	A fast, miniaturized, MCU-based board
Communications	A protocol-powered machine
Information Technology (IT)	A client
Electronics Industry	A development tool
Agriculture original equipment manufacturing (OEM)	A proprietary, closed, turn-key solution
Agricultural Science	A remote sensor/actuator
Farmers	An expensive telemetry equipment

**Table 2:** The seven conceptual functional layers of a WSAN hardware node.

Layer Level	Layer Name	Abbreviation
1	Data acquisition and Control Layer	DCL
2	Wireless Networking Layer	WNL
3	Data Gateway Layer	DGL
4	Application-Specific Layer	ASL
5	Programming and Debugging Layer	PDL
6	Power Management Layer	PML
7	Evaluation and Testing Layer	ETL

**Table 3:** Specifications of a 90 mm diameter PVC tube that can be used as the encapsulation and support pole of WSAN node according to the SensoTube architecture.

Parameter	Pressure Tolerance	
	4 atms	6 atms
Material	PVC-U	
Outer Diameter	90 mm	
Thickness	1.8 mm up to 2.2 mm	2.7 mm up to 3.2 mm
Mass	0.785 kg/m	1.15 kg/m
Standards	EN1452-2 [157], DIN 8061, DIN 8062	
Cost	4 USD/m	

**Table 4:** Current measurements in full active and in deep sleep states of the MCU of three different popular open-source hardware platforms.

Platform Name	Brand Name	*I_fa_* (mA)	*I_ds_* (mA)	*I_mcu_* = (*I_fa_* − *I_ds_*) (ma)
Arduino Uno Rev. 3	Arduino	46.85	33.17	13.68
Nucleo STM32L152	ST	56.95	50.24	6.71
FRDM-KL25Z	Freescale/NXP	36.06	34.16	1.90

**Table 5:** The parts list of the MCU circuitry of Arduino Uno Rev. 3.

#	Component	Description	Qty	Part Number	Manufacturer	Cost (euros)
1	C4	Capacitor	1	CC0603KRX7R9BB104	Yageo	0.091
2	C6	Capacitor	1	CC0603KRX7R9BB104	Yageo	0.091
3	28 pins IC Socket	Connector	1	1-390261-9	TE Conn	0.1
4	6 pins Header Fem.	Connector	1	5-534237-4	TE Conn	1.08
5	10 pins Header Fem.	Connector	1	M20-7821046	Harwin	0.73
6	8 pins Header Fem.	Connector	1	M20-7820842	Harwin	0.996
7	8 pins Header Fem.	Connector	1	M20-7820842	Harwin	0.996
8	XTAL1	Crystal	1	ABL-16.000MHZ-B2	Abracon	0.357
9	D2	Diode	1	CD1206-S01575	Bourns	0.142
10	ZU4	IC	1	ATMEGA328P-PU	Atmel	3.35
11	RN3	Resistor	1	CAY16-220J4LF	Bourns	0.095
12	RESET	Switch	1	TS42031-160R-TR-7260	Omron	0.317
					**Total:**	**8.345 euros**

**Table 6:** The parts list of the programming and debugging circuitry of Arduino Uno Rev. 3.

#	Component	Description	Qty	Part Number	Manufacturer	Cost (euros)
1	C7	Capacitor	1	CC0603KRX7R9BB104	Yageo	0.091
2	C5	Capacitor	1	CC0603KRX7R9BB104	Yageo	0.091
3	C8	Capacitor	1	GRM188R60J105KA01D	Murata	0.091
4	C11	Capacitor	1	500R14N220JV4T	Johanson	0.091
5	C9	Capacitor	1	500R14N220JV4T	Johanson	0.091
6	ICSP1	Connector	1	67996-406HLF	FCI	0.244
7	ICSP	Connector	1	67996-406HLF	FCI	0.244
8	X2	Connector	1	USB-B1HSW6	On Shore	0.58
9	XT1	Crystal	1	CSTCE16M0V53-R0	Murata	0.416
10	D3	Diode	1	CD1206-S01575	Bourns	0.142
11	L1	Ferrite	1	BLM21PG221SN1D	Murata	0.1
12	U3	IC	1	ATMEGA16U2-MUR	Atmel	3.93
13	RX	LED	1	APT2012YC	Kingbright	0.119
14	TX	LED	1	APT2012YC	Kingbright	0.119
15	F1	PTC	1	MF-MSMF050-2	Bourns	0.208
16	R1	Resistor	1	ERJ-3GEYJ105V	Panasonic	0.091
17	R2	Resistor	1	ERJ-3GEYJ105V	Panasonic	0.091
18	RN4	Resistor	1	CAY16-102J4LF	Bourns	0.095
19	Z1	Varistor	1	CG0603MLC-05E	Bourns	0.18
20	Z2	Varistor	1	CG0603MLC-05E	Bourns	0.18
					**Total:**	**7.194 euros**

**Table 7:** The parts list of the power management circuitry of Arduino Uno Rev. 3.

#	Component	Description	Qty	Part Number	Manufacturer	Cost (euros)
1	C1	Capacitor	1	CC0603KRX7R9BB104	Yageo	0.091
2	C2	Capacitor	1	CC0603KRX7R9BB104	Yageo	0.091
3	C3	Capacitor	1	GRM188R60J105KA01D	Murata	0.091
4	PC1	Capacitor	1	EMVA250ADA470MF55G	United Chemi-Con	0.32
5	PC2	Capacitor	1	EMVA250ADA470MF55G	United Chemi-Con	0.32
6	X1	Connector	1	PJ-102AH	CUI Inc	1.08
7	D1	Diode	1	S2M-13-F	Diodes Inc.	0.311
8	U1	IC	1	NCP1117ST50T3G	On Semi	0.398
9	U2	IC	1	LP2985-33DBVR	TI	0.498
10	U5	IC	1	LMV358IDGKR	TI	0.67
11	L	LED	1	APT2012YC	Kingbright	0.119
12	ON	LED	1	LG R971-KN-1	OSRAM	0.22
13	RN1	Resistor	1	CAY16-103J4LF	Bourns	0.095
14	RN2	Resistor	1	CAY16-102J4LF	Bourns	0.095
15	T1	Transistor	1	FDN340P	Fairchild	0.338
					**Total:**	**4.737 euros**

**Table 8:** The PDL shield’s four different JTAG connectors for the programming and debugging of the most popular MCUs for WSAN nodes.

Name	Description	Number of Pins	Supported MCUs/CPUs
P14	JTAG ARM	20 (2 × 10)	ARM, ARM-Cortex
P12	MSP430 JTAG	14 (2 × 7)	MSP430
P15	AVR JTAG ICE mkII	10 (2 × 5)	ATMEL AVR MCUs
P13	JTAG Spy-Bi-Wire	5 (1 × 5)	MSP430 MCUs

## Abbreviations

The following abbreviations are used in this manuscript:

WSAN	Wireless Sensors and Actuators Network
FPGA	Field-Programmable Gate Arrays
MCU	Microcontroller Unit
WMN	Wireless Multimedia Network
RFID	Radio Frequency Identification
ARM	Advanced RISC Machine
RISC	Reduced Instruction Set Computer
IoT	Internet of Thing
HMI	Human-Machine Interface
MEMS	Micro-Electromechanical Sensor
LoC	Lab-on-Chip
COTS	Commercial-Of-The-Shelve
NRE	Non-Recurring Engineering
OSH	Open-Source Hardware
API	Application Peripheral Interface
RF	Radio Frequency
PCB	Printed-Circuit Board
IC	Integrated Circuit
MPU	Microprocessor Unit
IDE	Integrated Development Environment
OSEP	Open-Source Expandable Platforms
SBC	Single Board Computer
BEC	Board Exoansion Connector
WuR	Wake-up-Radio
DCL	Data Acquisition and Control Layer
WNL	Wireless Networking Layer
DGL	Data Gateway Layer
ASL	Application-Specific Layer
PML	Power Management Layer
PDL	Programming and Debugging Layer
ETL	Evaluation and Testing Layer
JTAG	Joint Test Action Group
DAQ	Data Acquisition
SI	Signals Integrity
EMC	Electromagnetic Compatibility
EMI	Electromagnetic Interference
ESD	Electrostatic Discharge
IoG	Internet-of-Gateways
CPS	Cyber-Physical Space
SFSM	Synchronous Finite State Machine
ISP	In-System Programming
SMP	Symmetric Multicore Processing
AMP	Asymmetric Multicore Processing
RCP	Rapid Control Prototyping
MDE	Model-Driven Engineering
PVC	Poly-Vinyl Chloride
SPI	Serial Peripheral Interface
I2C	Inter-Integrated Communication
OTA	Over-The-Air
